# The Candidate Effector Cgmas2 Orchestrates Biphasic Infection of *Colletotrichum graminicola* in Maize by Coordinating Invasive Growth and Suppressing Host Immunity

**DOI:** 10.3390/ijms27020845

**Published:** 2026-01-14

**Authors:** Ziwen Gong, Jinai Yao, Yuqing Ma, Xinyao Xia, Kai Zhang, Jie Mei, Tongjun Sun, Yafei Wang, Zhiqiang Li

**Affiliations:** 1 Shenzhen Branch, Guangdong Laboratory of Lingnan Modern Agriculture, Genome Analysis Laboratory of the Ministry of Agriculture and Rural Affairs, Agricultural Genomics Institute at Shenzhen, Chinese Academy of Agricultural Sciences, Shenzhen 518120, China; gongzw91@163.com (Z.G.); suntongjun@caas.cn (T.S.); 2 State Key Laboratory for Biology of Plant Diseases and Insect Pests, Institute of Plant Protection, Chinese Academy of Agricultural Sciences, Beijing 100193, China; yaoja@163.com (J.Y.); myq15718881366@163.com (Y.M.); bioxxy@163.com (X.X.); zhangkai@tio.org.cn (K.Z.); mj1992yx@126.com (J.M.); 3 Fujian Key Laboratory for Monitoring and Integrated Management of Crop Pests, Fujian Engineering Research Center for Green Pest Management, Institute of Plant Protection, Fujian Academy of Agricultural Sciences, Fuzhou 350013, China; 4 Shandong Key Laboratory for Green Prevention and Control of Agricultural Pests, Institute of Plant Protection, Shandong Academy of Agricultural Sciences, Jinan 250100, China; 5 Key Laboratory of Marine Biogenetic Resources, Third Institute of Oceanography, Ministry of Natural Resources, Xiamen 361005, China; 6 College of Plant Protection, Henan Agricultural University, Zhengzhou 450002, China

**Keywords:** *Colletotrichum graminicola*, maize, effectors, ethylene, benzoxazinoids

## Abstract

Maize (*Zea mays* L.) is a major economic crop highly susceptible to *Colletotrichum graminicola*, the causal agent of anthracnose leaf blight, which causes substantial annual yield losses. This fungal pathogen employs numerous effectors to manipulate plant immunity, yet the functions of many secreted proteins during biphasic infection remain poorly characterized. In this study, we identified CgMas2, a candidate secreted protein in *C. graminicola* and a homolog of *Magnaporthe oryzae* MoMas2. Deletion of *CgMAS2* in the wild-type strain CgM2 did not affect fungal vegetative growth or conidial morphology but significantly impaired virulence on maize leaves. Leaf sheath infection assays revealed that CgMas2 is required for biotrophic invasive hyphal growth, as the mutant showed defective spreading of invasive hyphae to adjacent cells. Subcellular localization analysis indicated that CgMas2 localizes to the cytoplasm of conidia and to the primary infection hyphae. Furthermore, DAB staining demonstrated that disrupt of *CgMAS2* leads to host reactive oxygen species (ROS) accumulation. Comparative transcriptome analysis of maize infected with Δ*Cgmas2* versus CgM2 revealed enrichment of GO terms related to peroxisome and defense response, along with up-regulation of benzoxazinoid biosynthesis genes (benzoxazinone biosynthesis 3, 4 and 5) at 60 h post-inoculation (hpi). Conversely, six ethylene-responsive transcription factors (ERF2, ERF3, ERF56, ERF112, ERF115 and ERF118) involved in ethylene signaling pathways were down-regulated at 96 hpi. These expression patterns were validated by RT-qPCR. Collectively, our results demonstrate that CgMas2 not only promotes invasive hyphal growth during the biotrophic stage but may also modulate phytohormone signaling and defense compound biosynthesis during the necrotrophic phase of infection.

## 1. Introduction

The genus *Colletotrichum* comprises a group of fungal pathogens that attack a wide range of crops worldwide, including over 200 species which infect more than 720 host plants [1]. *C. graminicola* primarily infects maize, causing anthracnose leaf blight and stalk rot. These diseases lead to substantial annual yield losses globally, posing a serious threat to maize production and food security [2,3]. Therefore, investigating the molecular mechanisms of the *C. graminicola*-maize interaction is essential for developing effective disease management strategies.

*C. graminicola* employs a biphasic infection strategy, shifting from biotrophy to necrotrophy. The process begins when spores adhere to the host leaf surface, germinate, and form specialized appressoria that generate mechanical force to penetrate the host cuticle. Following penetration, the fungus develops bulbous biotrophic hyphae contained within the host plasma membrane, later differentiating into destructive necrotrophic hyphae that destroy host tissues [4,5]. To counteract such invasion, plants deploy a multi-layered defense system. This includes strengthened physical barriers like the cell wall and cuticle, as well as inducible chemical defenses, such as phytoalexins, defense-related hormones, and ROS. For example, overexpressing *ZmXYXT2* maize plants significantly alter cell wall composition, leading to cell wall thickening, thereby blocking intracellular invasion and colonization of *Fusarium verticillioides* [6]. In addition, OsNAC29, a key component of a MAPK signaling pathway in rice (*Oryza sativa*) disease resistance, binds directly to the CACGTG motifs in the promoters of *OsTPS28* and *OsCYP71Z2* to regulate the biosynthesis of the phytoalexin 5,10-diketo-casbene, thereby conferring blast resistance [7].

In the evolutionary arms race, pathogenic fungi have evolved to secrete effector proteins that suppress or manipulate these host defenses. These effectors can directly interfere with plant immune signaling or modify host cellular processes to facilitate infection [8]. For instance, the *Phytophthora sojae* effector PsFYVE1 targets the RNA-binding protein RZ-1A, which interacts with splicing factors such as NbGRP7, NbGRP8, and NbU1-70K, to suppress plant immunity [9]. Similarly, the *Ustilago maydis* effector Erc1 modifies host cell wall components to attenuate β-glucan-triggered defense and promote cell-to-cell movement in maize [10]. Although several effectors in *C. graminicola* have been identified, the majority of its effector repertoire and their precise functions during the biphasic infection cycle remain largely uncharacterized.

To comprehensively dissect these complex molecular dialogues, high-throughput transcriptional analysis has become an indispensable tool. RNA sequencing (RNA-seq) enables genome-wide, accurate quantification of gene expression and has been widely used to study plant response to biotic stress [11,12]. For example, transcriptome analysis of the maize inbred line MO17 infected with *Bipolaris zeicola* revealed significant reprogramming of plant hormone signaling and photosynthesis pathways [13]. The availability of high-quality maize genome sequences [14,15,16] further empowers such studies by providing essential references for data analysis.

Among the various fungal effector families, the Magnaporthe appressoria specific (Mas) protein family, first reported in 2002 [17], is of particular interest. In *Magnaporthe oryzae*, several proteins, including MoMas3, function as an apoplastic effector that not only contributes to fungal virulence but also suppresses rice immunity [18]. MoMas1, MoMas2 and MoMas5 are virulence factors of *M. oryzae* that specifically localizes to appressoria during infection [18]. *C. graminicola* possesses a homolog of MoMas2, designated CgMas2 (GLRG_03323), whose biological function is unknown. Given the critical of effectors in modulating plant immunity and the documented importance of Mas proteins in related fungi, we hypothesized that CgMas2 is a virulence effector in *C. graminicola*. To test this hypothesis and elucidate the role of CgMas2, we integrated fungal genetics, cell biology and transcriptomics. We generated ∆*Cgmas2* deletion mutant and complementation (CgMas2-GFP) strains and assessed their pathogenicity, morphological development, subcellular localization, and the consequent transcriptional changes in the host during infection. Our results demonstrate that while CgMas2 is dispensable for sporulation and vegetative growth, it is required for full virulence, likely through promoting invasive hyphal growth and modulating host defense and hormone signaling pathways. This study advances our understanding of the molecular interplay in the maize-*C. graminicola* pathosystem.

## 2. Results

### 2.1. CgMas2 Is Required for Full Virulence of C. graminicola

**Figure 1:**
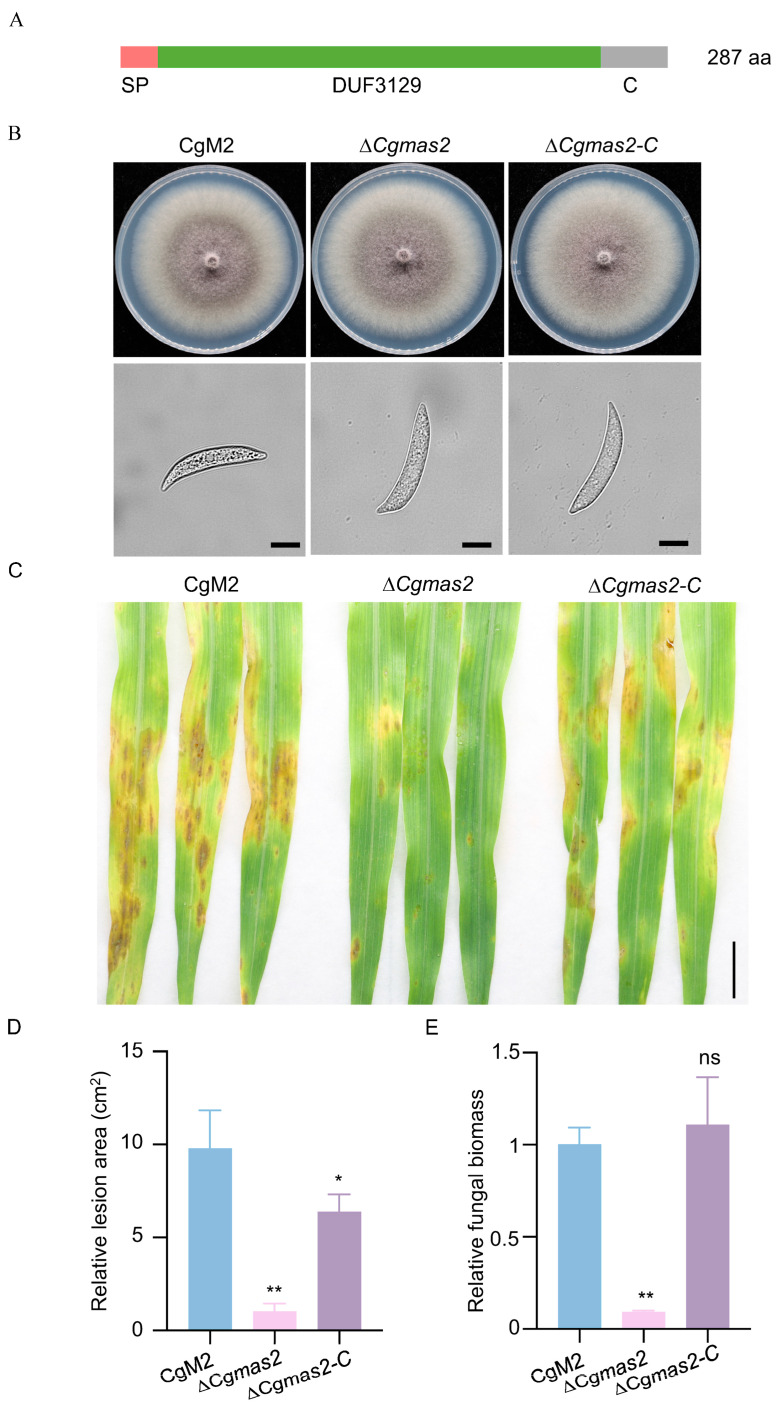
*CgMAS2* is required for the full virulence of *C. graminicola*. (**A**) Schematic diagram of the CgMas2 structural domain; SP represents the signal peptide, DUF3129 represents the domain with unknown function 3129, and C represents the C-terminus. (**B**) Deletion of *CgMAS2* does not affect the hyphal growth or conidial morphology of *C. graminicola.* The hyphal growth (**up panel**) and conidial morphology (**down panel**) of CgM2, ∆*Cgmas2* and ∆*Cgmas2-C* strains are shown. Scale bar is 20 µm. (**C**) Pathogenicity of *C. graminicola* is compromised in the ∆*Cgmas2* mutant. Conidial suspensions (5 × 10^5^/mL) of *C. graminicola* wild-type CgM2, *Cgmas2* mutant and ∆*Cgmas2-C* strains were sprayed onto two-week-old B73 maize seedlings at 25 °C for 5 days. The lesions were photographed at 5 dpi. Scale bar is 1 cm. (**D**,**E**) statistical analysis of the relative lesion area (**D**) and fungal biomass (**E**) on the leaves (*n* > 3) of B73 plants with CgM2, ∆*Cgmas2* mutant and ∆*Cgmas2-C* inoculation. The error bars indicate the standard deviations of three replicates; significance was analyzed via Student’s *t* test-unpaired *t* test (* *p* < 0.05, ** *p* < 0.01), and asterisks indicate significant differences, ns indicates no significant differences. All these experiments were performed with three independent biological repeats, and similar results were obtained.

Mas family proteins, including MoMas1, MoMas2, MoMas3 and MoMas5, are essential for the pathogenicity of *M. oryzae* [17,18]. However, the biological functions of their homologs in *C. graminicola* remain largely unknown. Using the sequences of MoMas proteins as queries, we identified six candidate homologs in *C. graminicola* via the NCBI database, designated CgMas1 to CgMas6 (Table S1). Among these, CgMas2, a 287-amino-acids protein, shared the highest identity (68.99%) with MoMas2 (Table S1). Phylogenetic analysis further supported a close evolutionary relationship between MoMas2 and CgMas2 (Figure S1A). We therefore selected CgMas2 for functional characterization. Conserved domain analysis predicted that CgMas2 contains an N-terminal signal peptide and a Domain of Unknown Function 3129 (DUF3129) (Figure 1A). To investigate the role of CgMas2, we generated Δ*Cgmas2* deletion mutants in the wild-type strain CgM2 using homologous recombination. All mutants were verified by PCR and RT-PCR using four primer pairs, yielding two positive strains (Δ*Cgmas2#8* and *#9*) (Figure S1B,C). The Δ*Cgmas2#8* was chosen for subsequent studies. We also generated the Δ*Cgmas2-C* complementation strains, a C-terminal green fluorescent protein (GFP) tagged version of the protein (CgMas2-GFP) was introduced into the Δ*Cgmas2* mutant background. Two independent transformants (CgMas2-GFP #1 and #3) expressed the fusion protein, as confirmed by Western blot analysis (Figure S1D). When cultured on complete medium (CM) and oatmeal medium (OM) plates, the Δ*Cgmas2*#8 showed no discernible differences from CgM2 and the Δ*Cgmas2-C* in colony morphology or conidial structure (Figure 1B), indicating that CgMas2 is not involved in vegetative growth or conidial morphology.

We next assessed the contribution of CgMas2 to pathogenicity. Two-week-old maize B73 seedlings were spray-inoculated with conidial suspensions of CgM2, Δ*Cgmas2* mutant and Δ*Cgmas2-C* strains. Disease symptoms evaluated at 5 days post-inoculation (dpi) revealed that leaves infected with Δ*Cgmas2* mutant developed significantly fewer and smaller lesions compared to those infected with CgM2 and Δ*Cgmas2-C* strains (Figure 1C). Quantification confirmed a significant decrease in relative lesion area in Δ*Cgmas2*-infected plants (Figure 1D). Consistent with this, fungal biomass was also lower in Δ*Cgmas2* infections than in wild-type CgM2 and Δ*Cgmas2-C* infections (Figure 1E). These results, reproducible across three independent experiments, demonstrate that *CgMas2* is required for the full pathogenicity of *C. graminicola*.

### 2.2. Functional Validation of the CgMas2 Signal Peptide

To verify the functionality of the predicted signal peptide, the sequences encoding the signal peptide (CgMas2-sp) and a truncated version without the signal peptide (CgMas2-ns) were fused to the pSUC2 vector and transformed into the yeast strain YTK12. In the yeast secretion trap assay, only yeast strains carrying pSUC2-CgMas2-sp or the positive control pSUC2-Avr1b grew on the selective YPRAA medium containing raffinose as the sole carbon source. In contrast, strains carrying pSUC2-CgMas2-ns, the negative control pSUC2-mg87, the empty pSUC2 vector, or the untransformed YTK12 strain failed to grow (Figure S2A). The secretion capability was further confirmed by a TTC reduction assay, in which only cultures of pSUC2-CgMas2-sp and pSUC2-Avr1b turned red due to the formation of insoluble triphenylformazan, whereas other constructs showed no color change (Figure S2B). These results confirm that CgMas2 may be a secreted protein with a functional signal peptide.

### 2.3. The Δ*Cgmas2* Mutant Is Defective in Biotrophic Growth

**Figure 2:**
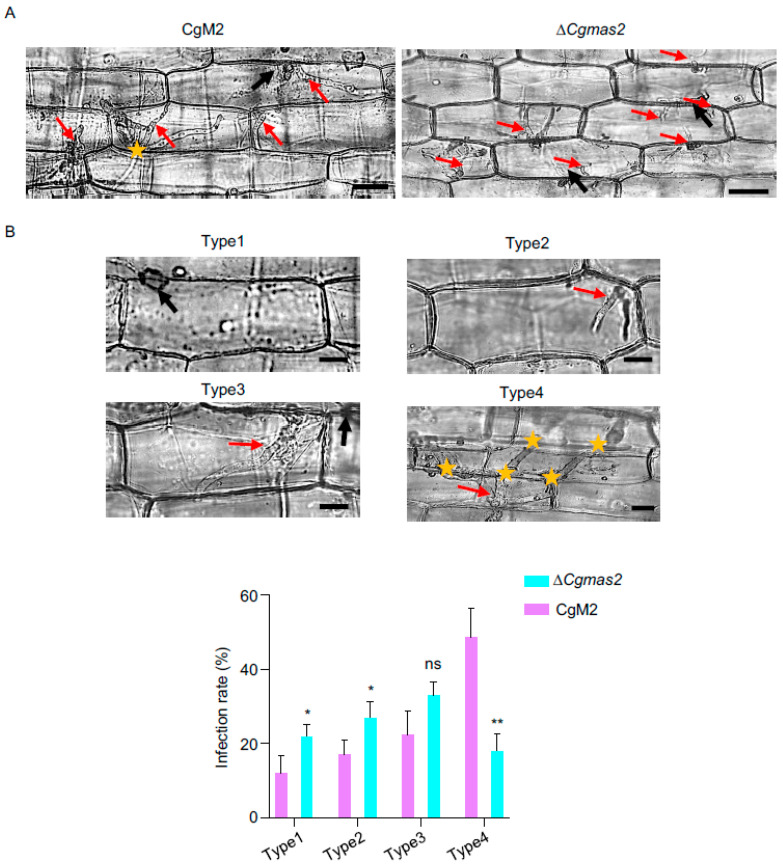
Single deletion of *CgMAS2* leads to limited invasive hyphae (IH). (**A**) A penetration assay was performed in a maize leaf sheath, and IH grown in maize cells was observed at 24 hpi. Scale bar is 20 µm. (**B**) Statistical analysis of each type of invasive hyphal shape for the indicated strains infecting the maize leaf sheath at 40 hpi. Scale bar is 10 µm. Five spots on each leaf sheath were randomly selected. Each spot contained at least approximately 50 infection sites, which were used to quantify different types of infection hyphae. Type 1, no penetration; type 2, a single or short primary hypha; type 3, IH expanded within a plant cell; type 4, IH invading neighboring cells. The error bars represent the standard deviations. significance was analyzed via multiple *t* test-unpaired *t* test (* *p* < 0.05, ** *p* < 0.01), ns means no significant differences and asterisks indicate significant differences. The black and red arrows indicate appressoria and invasive hyphae, respectively; and the yellow stars indicate invasive hyphae that moved into neighboring cells.

To investigate the cause of the reduced virulence in the Δ*Cgmas2* mutant, we performed a maize leaf sheath infection assay to compare the development of invasive hyphae between CgM2 and Δ*Cgmas2*. Live-cell imaging revealed that the infectious hyphae of the Δ*Cgmas2* mutant were shorter than those of the wild-type strain at 24 hpi (Figure 2A). Quantitative analysis at 40 hpi showed that approximately 30% and 20% of the infected sheath cells inoculated with Δ*Cgmas2* exhibited type 1 and type 4 infection patterns, respectively. In contrast, the wild-type CgM2 showed 10% type 1 and 50% type 4 (Figure 2B). These results indicate that CgMas2 is necessary for normal biotrophic growth of *C. graminicola* during host infection.

### 2.4. CgMas2 Localizes to the Conidia Cytoplasm and Initial Segment of the Primary Invasive Hyphae

**Figure 3:**
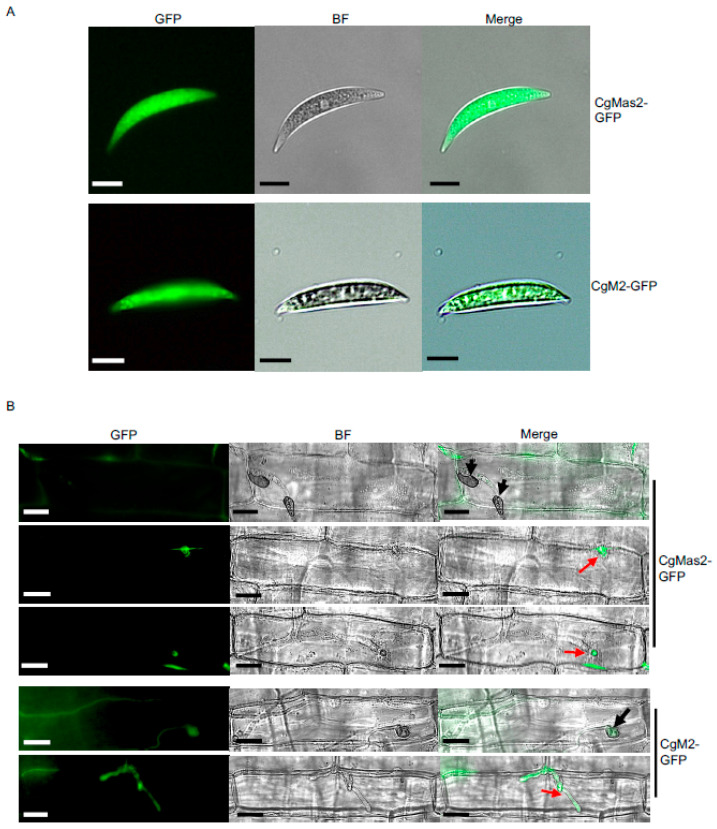
CgMas2 is expressed in the cytoplasm of conidia and primary bulbous hyphae. (**A**) Subcellular localization of CgMas2-GFP (**the up panel**) and CgM2-GFP (**the down panel**) in the conidia. Scale bar is 20 µm. (**B**) Live-cell imaging of maize leaf sheathes infected by CgMas2-GFP and the control CgM2-GFP strains showed the green fluorescence signals representing CgMas2 and CgM2-GFP localization, respectively, during infection. Scale bar is 20 µm. The black and red arrows indicate appressoria and invasive hyphae, respectively. Two CgMas2-GFP strains (#1 and #3) were used for the localization assay. Both exhibited similar localization patterns in conidia and during infection. Data from the representative strain CgMas2-GFP#1 are shown here. The experiments were performed with two independent biological repeats, and similar results were obtained.

To determine the subcellular localization of CgMas2 during fungal development, the CgMas2-GFP #1 strain was selected for detailed localization studies. We first examined CgMas2 localization in conidia germinating on an artificial hydrophobic surface. In these cells, the CgMas2-GFP signal was diffusely localized throughout the cytoplasm of conidia, a pattern similar to that of the cytosolic GFP control strain CgM2-GFP (Figure 3A). We next assessed its localization during host infection. Microscopic observation of infected maize leaf sheaths revealed that CgMas2-GFP signal specifically accumulated in the initial segment of primary invasive hyphae but was absent from the appressoria at 26 hpi (Figure 3B). In contrast, the control CgM2-GFP strain exhibited fluorescence distributed in both appressoria and throughout the infectious hyphae (Figure 3B).

### 2.5. Disruption of CgMAS2 Leads to Accumulation of ROS in Host

**Figure 4:**
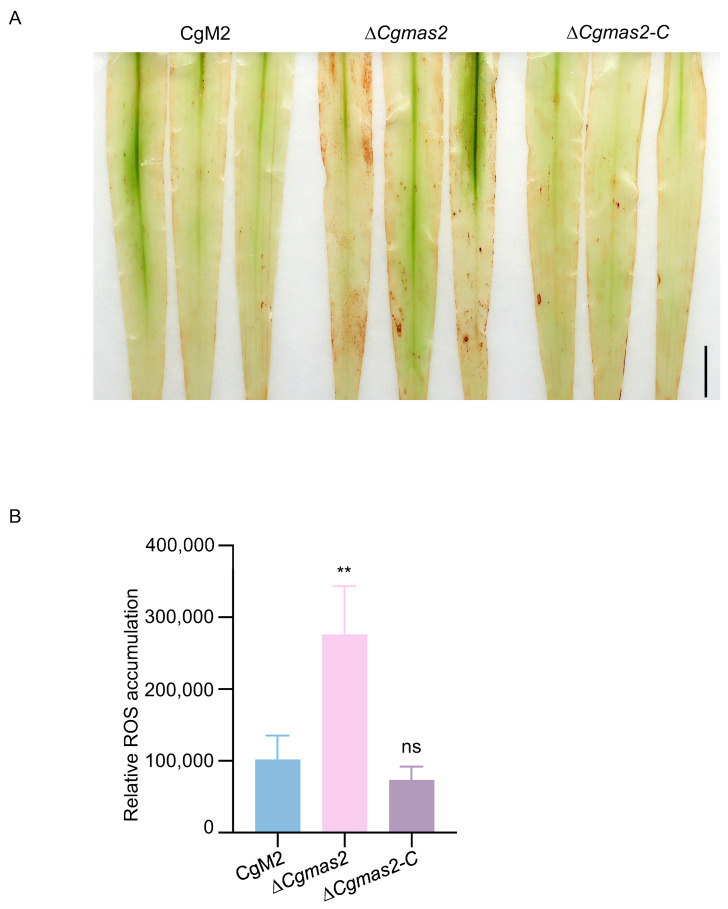
Deletion of *CgMAS2* leads to ROS accumulation in maize leaves. (**A**) DAB staining of maize leaves (*n* > 3) infected by CgM2, ∆*Cgmas2* mutant and ∆*Cgmas2-C* strains at 60 hpi. Scale bar is 1 cm. (**B**) Relative ROS accumulation was measured by Image J (version 1.52q). Error bars indicate the standard deviation. Significance was analyzed via Student’s *t* test-unpaired *t* test (** *p* < 0.01), and asterisks indicate significant differences, ns indicates no significant differences. These experiments were performed with two independent biological repeats, and similar results were obtained.

ROS are a well-established signature of plant responses to pathogen infection. To investigate the potential role of CgMas2 in modulating host immunity, we compared ROS accumulation in maize leaves infected with either CgM2 or the Δ*Cgmas2* mutant using 3,3′-diaminobenzidine (DAB) staining. Leaves inoculated with the Δ*Cgmas2* mutant showed intense brown polymerization, indicative of elevated ROS levels. In contrast, leaves infected with the wild-type CgM2 displayed significantly less DAB staining (Figure 4A,B). This finding suggests that CgMas2 may be involved in suppressing or scavenging pathogen-induced ROS burst during infection, a function consistent with its role in dampening host immunity.

### 2.6. The Differentially Expressed Genes in Maize Infected by CgM2 and ∆Cgmas2 Strain

**Figure 5:**
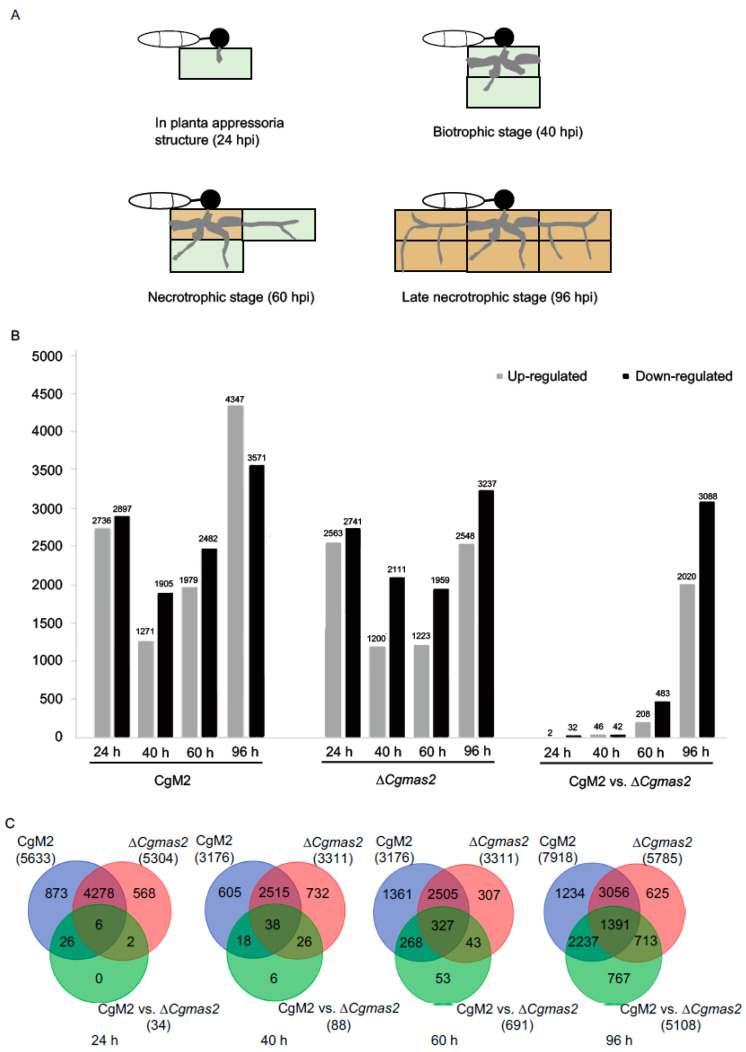
Comparison of differentially expressed genes (DEGs) in maize response to CgM2 and ∆*Cgmas2* infection at the different invasion stages. (**A**) schematic diagrams of four different infection stages of *C. graminicola* in maize. Light green and brown color rectangles represent alive and necrotic plant cells, respectively. (**B**) the histogram shows the number of up- and down-regulated DEGs in different comparisons. (**C**) Venn diagrams show unique and common DEGs in different comparisons at 24, 40, 60 and 96 h. The blue, red and green circles represent CgM2, *∆Cgmas2* and CgM2 vs. *∆Cgmas2* DEGs.

To elucidate how *CgMAS2* deletion alters host transcriptional responses, we conducted a comparative transcriptome analysis of maize leaves inoculated with either the CgM2 or the Δ*Cgmas2* mutant. Leaf samples were collected at 24, 40, 60, and 96 hpi, representing key stages of fungal infection (Figure 5A), with non-inoculated leaves (0 hpi) serving as mock controls. After stringent quality control, 65.83–103.37 million high-quality reads were obtained per sample, of which over 80% were uniquely mapped to the maize reference genome (Table S2). Principal component analysis (PCA) based on gene expression profiles showed clear separation between infected and mock-treated samples, confirming a substantial transcriptomic reprogramming induced by *C. graminicola* infection (Figure S3). Biological replicates clustered tightly, indicating high data reproducibility (Figure S4).

Differentially expressed genes (DEGs) were identified using the DESeq2 package. We analyzed four temporal comparisons for each strain relative to mock (CgM2 vs. mock and Δ*Cgmas2* vs. mock at each time point), as well as direct comparisons between the mutant and wild-type at the same time points (e.g., Δ*Cgmas2* 24 hpi vs. CgM2 24 hpi). Histograms summarize the numbers of up- and down-regulated DEGs across these twelve comparisons (Figure 5B). Notably, the number of DEGs specifically altered in the Δ*Cgmas2* infection relative to wild-type infection increased dramatically over time, from 34 at 24 hpi to 5108 at 96 hpi (Figure 5C; all DEGs are listed in Table S3). These results demonstrate that infection by both strains triggers extensive transcriptional changes in maize, and that the absence of *CgMAS2* leads to a distinct and increasingly divergent host gene expression profile as infection progresses.

### 2.7. Gene Ontology Enrichment Analysis

To characterize the functional changes associated with CgM2 and Δ*Cgmas2* infections, we performed Gene Ontology (GO) enrichment analysis on the DEGs. At 24 hpi, up-regulated DEGs in infected leaves were enriched for processes such as “disaccharide biosynthetic process,” “trehalose biosynthetic process,” and “protein localization to organelle,” suggesting early host cell wall and metabolic reprogramming (Figure S5 and Table S4). By 40 hpi, during the biotrophic phase, enrichment shifted strongly toward “translation,” “ribosome,” and “protein folding,” indicating a broad upregulation of cellular biosynthesis in response to pathogen challenge. The transition to necrotrophy (around 60 hpi) revealed a marked divergence between strains. In Δ*Cgmas2*-infected leaves, up-regulated DEGs were enriched in defense-related terms including “response to bacterium” and “defense response.” In contrast, CgM2 infection at this stage induced terms “heme binding,” “monooxygenase activity,” and “oxidoreductase activity.” This divergence became more pronounced at 96 hpi: CgM2-infected leaves showed enrichment in “autophagy” and “cellular catabolic process,” whereas Δ*Cgmas2*-infected leaves remained enriched in “translational and antibacterial response terms” (Figures S5 and S6 and Table S4).

Conversely, down-regulated DEGs in both infections were consistently associated with chloroplast components and photosynthetic processes (“plastid part,” “photosynthesis,” “photosystem”) across multiple time points (Figure S7 and Table S5). Collectively, this temporal GO analysis reveals that infection by the Δ*Cgmas2* mutant elicits a stronger and more sustained host defense transcriptome compared to wild-type infection.

### 2.8. KEGG Enrichment Analysis

To delineate the specific metabolic and signaling pathways modulated during infection, we performed Kyoto Encyclopedia of Genes and Genomes (KEGG) enrichment analysis on up- and down-regulated DEGs (adjust *p* value < 0.05). A set of pathways was commonly enriched among up-regulated DEGs in both CgM2- and Δ*Cgmas2*-infected samples, including “valine, leucine and isoleucine degradation,” “circadian rhythm–plant,” “ribosome,” “peroxisome,” and “plant–pathogen interaction,” suggesting their general importance in the maize response to *C. graminicola* (Table S6).

Notably, several pathways displayed distinct enrichment patterns depending on the infection stage and fungal strain. In CgM2 infections, pathways such as “ribosome biogenesis in eukaryotes” and “spliceosome” were specifically up-regulated during early (appressorial) stages, while “fatty acid metabolism,” “beta-alanine metabolism,” and “autophagy” were uniquely enriched at later time points. In contrast, Δ*Cgmas2* infections triggered specific up-regulation of “starch and sucrose metabolism” at the appressorium stage, “photosynthesis–antenna proteins” during biotrophy, and “diterpenoid biosynthesis” along with “amino sugar and nucleotide sugar metabolism” in the necrotrophic phase (Table S6). Conversely, pathways related to “photosynthesis,” “carbon fixation,” “carbon metabolism,” and “biosynthesis of cofactors” were consistently down-regulated in both infection types, indicating a broad suppression of primary metabolism in infected leaves (Table S6).

**Figure 6:**
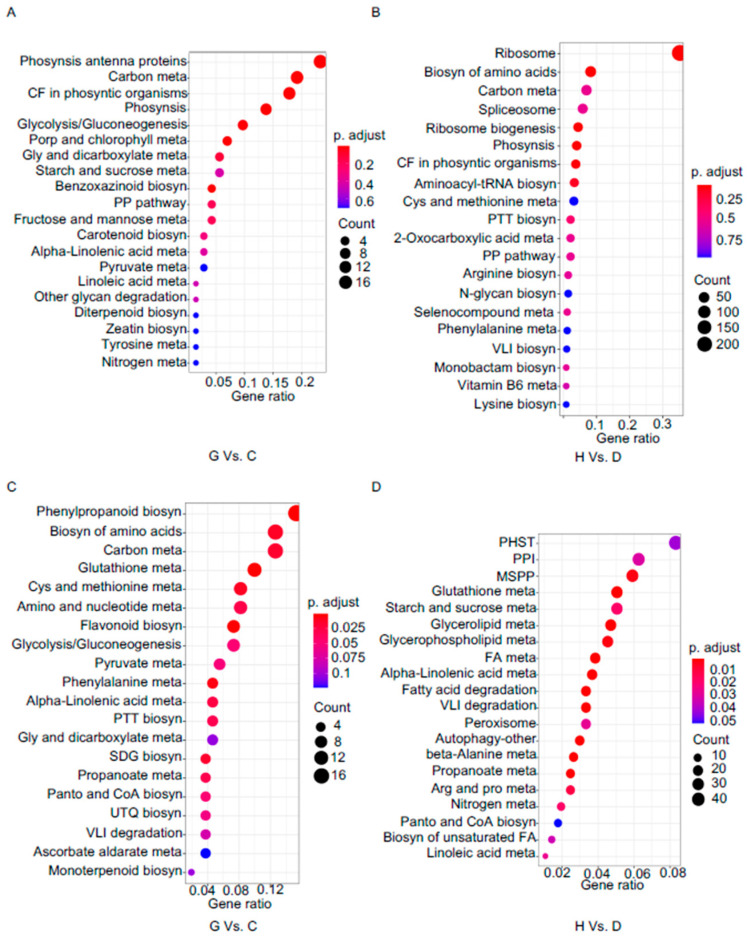
KEGG enrichment analysis in the comparison groups of C vs. G and D vs. H. (**A**,**B**) KEGG enrichment analysis was conducted on the upregulated DEGs of C vs. G (**A**) and D vs. H (**B**). (**C**,**D**) KEGG enrichment analysis was performed on the downregulated DEGs of C vs. G (**C**) and D vs. H (**D**). C vs. G indicate CgM2 60 hpi vs. ∆*Cgmas2* 60 hpi, D vs. H indicate CgM2 96 hpi vs. ∆*Cgmas2* 96 hpi. Phosynsis, Photosynthesis; meta, metabolism; CF, Carbon fixation; Phosyntic, Photosynthetic; Porp, Porphyrin; Gly, glyoxylate; Biosyn, biosynthesis; PP, pentose phosphate; Cys, Cysteine; PTT, Phenylalanine, tyrosine and tryptophan; PP, Pentose phosphate; VLI, Valine, leucine and isoleucine; SDG, Stillbenoid, diaryheptanoid and gingerol; Panto, Pantothenate; UTQ, Ubiquinone and other terpenoid-quinone; PHST, Plant hormone signal transduction; PPI, Plant-pathogen interaction; MSPP, MAPK signaling pathway-plant; FA, fatty acid; Arg and pro, Arginine and proline.

We further focused on pathways differentially regulated between Δ*Cgmas2* and CgM2 infections. At 60 hpi, up-regulated DEGs in the mutant-infected leaves were enriched in “photosynthesis–antenna proteins,” “benzoxazinoid biosynthesis,” and “diterpenoid biosynthesis” (Figure 6A), while down-regulated DEGs were enriched in “phenylpropanoid biosynthesis,” “biosynthesis of amino acids,” and “carbon metabolism” (Figure 6C). By 96 hpi, up-regulated DEGs showed enrichment in “biosynthesis of amino acids,” “spliceosome,” and “photosynthesis” (Figure 6B). Interestingly, “plant hormone signal transduction” and “plant-pathogen interaction” pathways were enriched among down-regulated DEGs at this late stage (Figure 6D). Together, these results highlight that infection with the Δ*Cgmas2* mutant elicits a stronger and more sustained activation of defense-related pathways (e.g., benzoxazinoid and diterpenoid biosynthesis) and a distinct repression of key signaling pathways (e.g., plant hormone transduction). This differential regulation likely contributes to the enhanced resistance observed in maize against the ΔCgmas2 strain during necrotrophic growth.

### 2.9. Benzoxazinone Biosynthesis Genes Were Up-Regulated in ∆Cgmas2-Infected Maize Leaves

**Figure 7:**
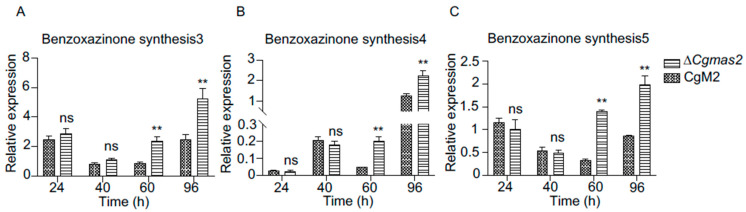
Expression of benzoxazinone synthesis genes were up-regulated at the late infection stage. (**A**–**C**) Transcript levels of benzoxazinone synthesis3 (**A**), 4 (**B**) and 5 (**C**) in CgM2- and ∆*Cgmas2*-infected plant tissue at different time points. Three independent assays were performed, and similar results were obtained. The error bars indicate the standard deviations of three replicates; significance was analyzed via Student’s *t* test-unpaired *t* test (** *p* < 0.01), ns means no significant differences and asterisks indicate significant differences.

Benzoxazinones and benzoxazolinones, which play key roles in allelopathy and as defense compounds against (micro) biological threats, are ubiquitous in Poaceae, such as wheat (*Triticum aestivum*) and maize. Among the up-regulated DEGs in ∆*Cgmas2* vs. CgM2 comparison at 60 hpi, we identified three key genes (benzoxazinone biosynthesis 3, 4, and 5) involved in benzoxazinoid biosynthesis. To validate their expression patterns, we performed RT-qPCR on maize leaves infected with the ∆*Cgmas2* or CgM2 across multiple time points. Both RNA-seq data and RT-qPCR confirmed that transcript levels of benzoxazinone biosynthesis 3, 4, and 5 were significantly higher in ∆*Cgmas2*-infected leaves at 60 hpi than in CgM2-infected leaves (Figure 7A–C). These results indicate that benzoxazinoid biosynthesis is specifically activated in response to infection by the *CgMAS2* deletion mutant, suggesting that this pathway contributes to maize defense when CgMas2 function is absent.

### 2.10. Most of the Genes Related to the Ethylene Signaling Pathways Were Down-Regulated

**Figure 8:**
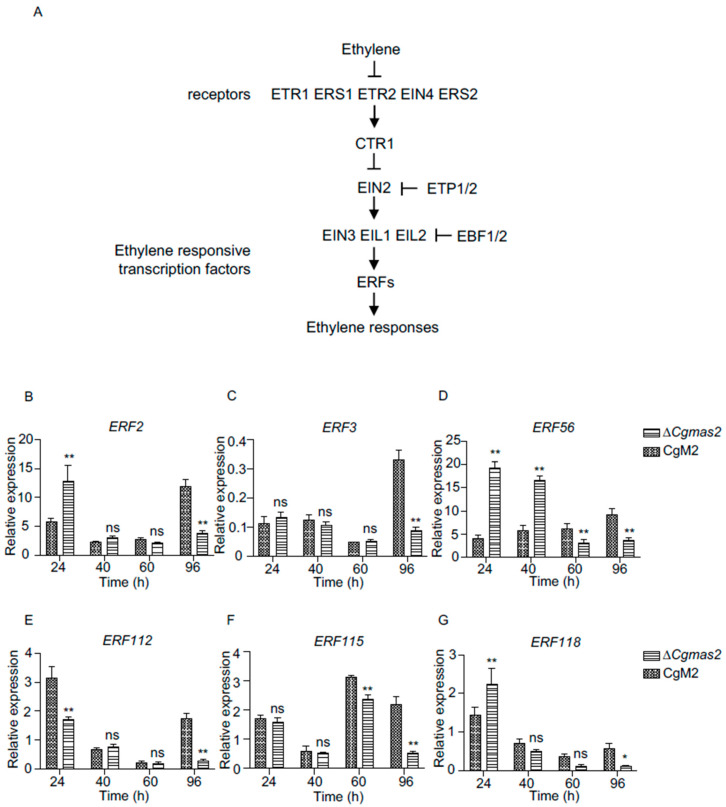
Genes related to the ethylene pathway were downregulated in ∆*Cgmas2*-infected plant tissue at 96 hpi. (**A**) the canonical ethylene signaling pathway in plants. (**B**–**G**) RT-qPCR was used to verify the transcript levels of ethylene responsive transcription factor 2 (ERF2) (**B**), ERF3 (**C**), ERF56 (**D**), ERF112 (**E**), ERF115 (**F**) and ERF118 (**G**) in CgM2 and ∆*Cgmas2*-infected maize leaves at different time points. Three independent assays were performed, and similar results were obtained. The error bars indicate the standard deviations of three replicates; significance was analyzed via multiple *t* test-unpaired *t* test (* *p* < 0.05, ** *p* < 0.01), ns means no significant differences and asterisks indicate significant differences.

Accumulating evidence has demonstrated that phytohormones, also known as plant hormones, often function in a complex and integrated manner to modulate plant immune responses against various pathogens. Notably, many genes associated with the ethylene (ET) signaling pathway, as listed in Table S7, were down-regulated among the differentially expressed genes (DEGs) in the Δ*Cgmas2* versus CgM2 comparison at 96 h post-inoculation (hpi) (Figure 6D). RNA-seq analysis revealed that fifteen out of eighteen ET-related genes showed decreased expression based on log_2_ fold-change data. These include genes encoding ethylene-responsive transcription factors such as ERF1A, ERF2, ERF3, ERF4, ERF55, ERF56, ERF112, ERF115, ERF118, RAP2-2, RAP2-3, an ethylene-responsive transcription factor-like protein, an AP2-like ethylene-responsive transcription factor, Reversion-to-Ethylene-Sensitivity1-like3, and an Ethylene-Insensitive3-like3 protein are downstream of ET signaling pathway (Figure 8A). In contrast, only three genes, namely EIN3-binding F-box protein 1, ERF1B, and an ethylene response factor, were up-regulated in the Δ*Cgmas2* vs. CgM2 group at 96 hpi (Tables S3 and S7).

To validate these findings, we randomly selected six downstream genes of the ET pathway, which included ERF2, ERF3, ERF56, ERF112, ERF115, and ERF118, and measured their transcript levels at different time points using RT-qPCR (Figure 8B–G). The results indicated that the expression of all six genes was reduced in Δ*Cgmas2*-infected samples at 96 hpi, consistent with the RNA-seq data (Table S7). These results suggest that the ET pathway may negatively regulate maize immunity in response to Δ*Cgmas2* infection. In summary, our data indicate that the secreted effector CgMas2 not only supports biotrophic growth but also may modulate plant hormone pathways and defense compound biosynthesis to promote infection during the necrotrophic phase.

## 3. Discussion

Maize is the most extensively cultivated cereal crop and achieves the highest yield in China. The hemibiotrophic ascomycete fungus *C. graminicola* causes maize anthracnose leaf blight, leading to substantial production and economic losses. Our results indicated that CgMas2 contains a functional signal peptide, a characteristic typical of candidate secreted proteins (Figure S2), although direct biochemical evidence of its secretion remains to be obtained. We generated a Δ*Cgmas2* mutant via homologous recombination. This mutant exhibited normal colony growth and conidial morphology but showed markedly reduced pathogenicity on maize leaves (Figure 1). Furthermore, a maize leaf sheath penetration assay demonstrated that CgMas2 is critical for hyphal growth during the biotrophic stage (Figure 2). These findings are consistent with reports on its homologs in *M. oryzae,* the MoMas proteins. For instance, MoMas1 (Gas1) and MoMas2 (Gas2) are required for full pathogenicity in *M. oryzae* but do not affect vegetative growth or sporulation [17]. Similarly, MoMas3, a secreted effector, does not influence colony growth or conidiation yet contributes to pathogenicity by suppressing *PR* gene expression and ROS accumulation, thereby facilitating infection [18]. In line with this mechanistic role, we found that infection by the Δ*Cgmas2* mutant triggered higher ROS accumulation in maize leaves compared to CgM2 and Δ*Cgmas2-C* infections (Figure 4). Collectively, our data suggest that CgMas2 promotes pathogen infection by modulating host ROS accumulation.

Although CgMas2 shared the highest protein identity (68.99%) with its *M. oryzae* homolog MoMas2 (Figure S1 and Table S1), their subcellular localization patterns during infection differ markedly. In this study, CgMas2 was found to localize specifically to the initial segment of the primary invasive hyphae at the early infection stage (Figure 3B), whereas MoMas2 is reported to accumulate in appressoria [17]. This divergence extends to other Mas family members: both MoMas3 and MoMas5 also localize to appressoria initially but later redistribute to the extra-invasive hyphal membrane compartment and penetration peg, respectively [18]. These distinct localization patterns suggest that Mas proteins may perform stage-specific or spatially segregated functions, possibly through interactions with different host targets. The molecular mechanisms by which CgMas2 contributes to pathogenicity in maize, including its precise host interactors and mode of action, remain to be elucidated.

To elucidate how maize responds to *C. graminicola* infection and how the effector CgMas2 manipulates plant immunity, we conducted a comparative transcriptome analysis. Notably, GO and KEGG enrichment analyses revealed a distinct transcriptional reprogramming in maize infected with the Δ*Cgmas2* mutant. In particular, pathways and terms related to photosynthesis, including “photosynthesis,” “thylakoid,” and “photosynthesis-antenna proteins” were notably upregulated at 60 hpi in Δ*Cgmas2*-infected leaves compared to CgM2-infected leaves (Figure 6A, Figures S5 and S6A). This finding contrasts with the typical suppression of photosynthetic genes observed during pathogen infection, as reported in previous studies [19]. *C. graminicola* infection causes necrotic lesions that directly damage leaf tissue and chloroplasts, the central organelles for photosynthesis. Beyond their role in carbon fixation, chloroplasts are essential for synthesizing amino acids, hormones, vitamins, secondary metabolites, and lipids, all of which contribute to plant defense signaling [20]. Moreover, chloroplasts are key sites for the generation of ROS. During photosynthetic electron transport, photosystem I (PSI) and photosystem II (PSII) produce superoxide, hydrogen peroxide and singlet oxygen, which can subsequently activate defense-related genes and hypersensitive responses under biotic stress [21]. The upregulation of photosynthetic components in the maize infected with the Δ*Cgmas2* mutant may indicate either a retained capacity for defense-related metabolism and ROS signaling, or it may be an indirect result of defense resource reallocation. Elucidating the precise relationship between CgMas2 and photosynthetic regulation presents an intriguing direction for future research.

Moreover, growing evidence indicates that pathogens often secrete effectors to suppress plant immunity by directly targeting chloroplast-localized proteins. For instance, the *Pseudomonas syringae* effector HopI1 disrupts thylakoid structure and inhibits salicylic acid biosynthesis to promote infection [22]. Similarly, the oomycete *Phytophthora brassiceae* secretes an effector that interacts with the chloroplast protein RPH1, which is required for activating specific immune responses against this pathogen [23]. In line with these observations, our transcriptome data revealed significant enrichment of the “benzoxazinoid biosynthesis” pathway in maize infected with the Δ*Cgmas2* mutant compared to the CgM2 at 60 hpi (Figure 6A). Benzoxazinoids, including benzoxazinones and benzoxazolinones, are specialized defense metabolites produced by species in the Poaceae family, such as maize, wheat, and rye [24]. These defense metabolites induced by biotic challenges and Insect herbivory [25,26,27]. The biosynthesis of major benzoxazinoid (BZX) compounds in maize is well characterized, involving 14 BX enzymes that convert indole-3-glycerol phosphate into more than 20 structurally diverse BZXs. In this pathway, indole produced by BX1 is first converted into indolin-2-one by the indole-2-monooxygenase BX2, and DIMBOA is subsequently generated from indolin-2-one through the action of BX3-BX5 [27,28]. A series of mechanistically diverse BX enzymes then catalyze further modifications, yielding various glycosylated hydroxamic acids such as DIMBOA-Glc, HDMBOA-Glc, and HDM2BOA-Glc (Figure S8). Recent studies have further elucidated the regulatory dynamics of these compounds. For example, volatile indole can be metabolized into benzoxazinoids, a conversion that partly contributes to its priming effect on maize defense, although indole also functions through BZX-independent signaling pathways [28]. Concurrently, a novel regulatory node in maize chemical defense has been identified: the nucleocytoplasmic protein phosphatase 2C, ZmPP2C45, negatively regulates benzoxazinoid accumulation and insect resistance by modulating the phosphorylation state of the transcription factor ZmBELL4 [27]. Our RT-qPCR analysis showed that transcript levels of benzoxazinone biosynthesis3-5 (BX3–BX5) were upregulated in maize infected with the Δ*Cgmas2* mutant at 60 and 96 hpi (Figure 7A–C), suggesting a corresponding increase in DIMBOA accumulation. Collectively, these findings imply that CgMas2 may promote infection by interfering with plant immunity, potentially through modulating the production of defense metabolites such as benzoxazinoids or by influencing related processes like photosynthesis.

Notably, transcriptome analysis revealed that plant hormone pathways were down-regulated in maize infected with the Δ*Cgmas2* compared to the CgM2 at 96 hpi (Figure 6D). This suppression was particularly evident for genes within the ET signaling pathway (Tables S3 and S7). In plants, canonical ET signaling pathway initiates with ET binding to its receptors, leading to conformational changes that reduce the interaction between CTR1 and EIN2 and decrease EIN2 phosphorylation. This reduction promotes EIN2 accumulation, as EIN2 is otherwise targeted for degradation by the F-box proteins ETP1 and ETP2 [29]. EIN2 subsequently activates EIN3-dependent transcription and the related transcription factor EIL1, thereby inducing ET responses (Figure 8A) [30]. The EIN3 and EIL1 proteins are degraded by the F-box proteins EBF1 and EBF2, which repress ethylene signaling [31]. Beyond its role in development, ET is a key modulator of plant immunity against diverse pathogens [32]. Pathogens often secrete effectors to interfere with this pathway and subdue host immunity. For example, in tomato, the *Xanthomonas euvesicatoria* type III effector XopD directly interacts with the ET-induced transcription factor SlERF4 to inhibit ET biosynthesis and promote virulence [33]. In our study, RT-qPCR validation aligned with RNA-seq data, showing a significant downregulation of six *ERF* genes upon infection with the Δ*Cgmas2* mutant at 96 hpi (Figure 8B–G). These results suggest that the absence of CgMas2 compromises the host ethylene response. However, direct measurement of ethylene levels during infection is needed to strengthen this transcriptional evidence. We therefore propose that CgMas2 may promote infection during the necrotrophic phase by modulating host ethylene signaling. The precise molecular mechanism by which CgMas2 interfaces with the ET pathway to dampen immunity warrants further investigation. In summary, our study demonstrates that the *C. graminicola* effector CgMas2 supports biotrophic invasion by facilitating invasive hyphal growth and likely contributes to necrotrophic colonization by manipulating host defense metabolism and hormone signaling.

## 4. Materials and Methods

### 4.1. Fungal Strain and Culture Conditions

The wild-type strain CgM2 was used as the parent strain to generate Δ*Cgmas2* mutants in this study. All fungal strains were cultured on complete medium plates, containing 6 g yeast extract, 3 g casamino acids, 3 g peptone, 10 g sucrose, and 15 g agar per liter of distilled water, for vegetative growth at 25 °C in darkness. For sporulation, strains were grown on oatmeal (OM) plates (30 g oatmeal and 15 g agar per liter distilled water) under a 12/12 h light/dark cycle at 25 °C. For liquid cultures, strains were inoculated into SY medium (171.15 g sucrose and 1 g yeast extract per liter of distilled water) and shaken at 25 °C for 40 h to generate mycelia for protoplast preparation using lysing enzymes. Mutant transformants were selected on TB3 agar (3 g yeast extract, 3 g casamino acids, 200 g sucrose, and 15 g agar per liter of distilled water) supplemented with 300 µg/mL hygromycin B.

### 4.2. Deletion of CgMAS2 Gene

The *CgMAS2* gene was disrupted using a split-PCR strategy for gene replacement, as previously described [18]. Briefly, approximately 1 kb upstream and downstream flanking sequences of the *CgMAS2* gene were amplified using primer pairs 1F/2R and 3F/4R, respectively. The hygromycin B phosphotransferase (HPH) cassette was amplified from a template using primers HYG-F/HYG-R. These three fragments were fused via double-joint PCR, and the resulting fusion product was transformed into CgM2 protoplasts. Putative knockout mutants were first selected on TB3 agar with hygromycin B and then confirmed by PCR using specific primer sets (OF/OR, UA/HYG-R1, and HYG-F1/DB).

### 4.3. Analysis of Colony and Conidial Morphology

To assess growth and development, the indicated strains were point-inoculated on CM or OM plates and incubated at 25 °C under appropriate light conditions for sporulation or darkness for vegetative growth. Colony morphology of the CgM2, Δ*Cgmas2* and Δ*Cgmas2-C* strains were photographed at 6 dpi. Conidia were harvested from OM plates at 7 dpi, and their morphology was examined and captured using a Leica microscope with bright-field optics.

### 4.4. Yeast Secretion Trap Assay

The presence of a signal peptide in CgMas2 was predicted using SignalP-5.0 [34]. The sequence encoding the predicted signal peptide of CgMas2 (CgMas2-sp) and a version lacking this sequence (CgMas2-NS) were cloned into the pSUC2 vector. These constructs were then transformed into the yeast strain YTK12. Transformed yeasts were grown on SD/-Trp medium and subsequently transferred to YPRAA medium containing 2 µg/mL antimycin A to assay for invertase secretion. The pSUC2-Avr1b and pSUC2-mg87 constructs served as positive and negative controls, respectively. Secretion activity was further confirmed by the reduction of 2,3,5-triphenyltetrazolium chloride (TTC) to red triphenylformazan, as described by Yin et al. [35].

### 4.5. Plant Material and Spray Inoculation

Maize seedlings were cultivated in a greenhouse under a 16 h photoperiod, with temperatures set to 28 °C during the day and 25 °C at night. Two-week-old seedlings of uniform size were selected for inoculation. Conidial suspensions of CgM2, Δ*Cgmas2* mutant and Δ*Cgmas2-C* were harvested from OM plates, washed with 0.05% Tween 20 solution, and adjusted to a concentration of 5 × 10^5^ spores/mL. The spore suspension was sprayed evenly onto the leaf surfaces. Inoculated plants were sealed in a humidity chamber and kept in darkness at 25 °C for the first 24 h. They were then transferred to a chamber with a 12/12 h light/dark cycle at 25 °C for 4 to 5 days until disease lesions developed. Infected leaves were then collected and photographed for analysis.

### 4.6. Maize Leaf Sheath Infection Assay

Maize B73 seeds were sown directly in soil-filled pots and grown in a greenhouse under a 12/12 h light/dark cycle for 5 days. An inoculation chamber was prepared with moist paper towels and supporting test tubes. A transparent leaf sheath was carefully peeled from the seedling and placed horizontally between two tubes. A spore suspension of the test strain was injected into the cavity of three individual leaf sheaths per replicate, ensuring no leakage. The chamber was incubated in darkness at 25 °C. Infection progression and invasive hyphal growth were monitored under a light microscope at designated time points. The experiment included three independent biological replicates. For statistical analysis of the infection rate, five spots on each leaf sheath were randomly selected. Each spot contained at least approximately 50 infection sites, which were used to quantify different types of infection hyphae.

### 4.7. EGFP Construct Preparation

To generate the CgMas2-EGFP fusion construct, the coding sequence (CDS) of *CgMAS2* (excluding the stop codon) was amplified using primers Np-F/GFP-R. The PCR product, containing 15–20 bp homology arms to the pGTN vector, was purified and fused with the linearized pGTN vector (digested with KpnI and HindIII) using homologous recombination. The reaction mixture was introduced into *E. coli* DH5α competent cells, which were then screened on ampicillin-containing plates. Positive clones were verified by colony PCR and subsequent DNA sequencing.

### 4.8. Sample Preparation, Library Construction, and RNA-Seq Analysis

Maize plants were inoculated via the spray method for transcriptome sampling. Leaf samples were collected at 0 h (uninoculated control), 24 h (appressorium formation stage), 40 h (biotrophic stage), 60 h (necrotrophic stage), and 96 h (late lesion formation stage), immediately frozen in liquid nitrogen, and stored at −80 °C. For each time point, a sample comprising at least five leaves from the same position was collected, with three biological replicates per condition. Equal amounts of total RNA from *C. graminicola*-infected and mock-inoculated leaves were used for RNA sequencing. Sequencing libraries were prepared from approximately 1 µg of total RNA per sample using the NEBNext^®^ Ultra™ RNA Library Prep Kit (New England Biolabs Inc., E7350, Ipswich, MA, USA), with index codes added to attribute sequences to each sample. Library preparation and sequencing on the Illumina HiSeq 2500 system (Illumina Inc., Hiseq2500, San Diego, CA, USA) were performed by Beijing Novo Gene Bioinformatics Technology Co., Ltd. (Beijing, China). For the quality control: Raw data (raw reads) of fastq format were first processed through fastp software (version 0.19.7, with fastp -g -q 5 -u 50 -n 15 -l 150 sets). In this step, clean data (clean reads) were obtained by removing reads (containing ploy-N and low-quality reads from raw data). At the same time, Q20, Q30 and GC content the clean data were calculated. All the downstream analyses were based on the clean data with high quality.

### 4.9. Identification and Enrichment Analysis of Differentially Expressed Genes

Differential gene expression analysis was performed using the DESeq2 R package (1.42.1) [36]. The *p*-values obtained were adjusted for multiple testing using the Benjamini and Hochberg method to control the false discovery rate (FDR) [37]. Genes exhibiting a fold change greater than or equal to 2 and an FDR less than 0.01 were classified as differentially expressed genes (DEGs). Gene Ontology (GO) enrichment analysis of DEGs was conducted using the GOseq R package (version 1.56.0), with terms having a corrected *p*-value below 0.05 considered significantly enriched [38]. KEGG pathway enrichment analysis was performed using KOBAS (accessed on 21 May 2021) [39], and pathways with a corrected *p*-value less than 0.05 were deemed significantly enriched.

### 4.10. Validation of DEGs by qRT-PCR

The expression levels of selected genes identified by RNA-seq were validated using quantitative reverse transcription PCR (RT-qPCR). Total RNA (2 µg) from different samples was reverse-transcribed into cDNA using M-MLV reverse transcriptase with random hexamer primers. Gene-specific primers were designed using Primer Premier software (version 5.0). The maize *actin1* gene was used as an internal reference for normalization. The qPCR amplification program consisted of an initial denaturation at 95 °C for 30 s, followed by 40 cycles of 95 °C for 5 s and 60 °C for 30 s. A melt curve analysis was performed post-amplification to verify reaction specificity. The reactions were carried out using 2 x SYBR Green Mix (Genestar, A304, Shanghai, China) on a Real-time qPCR instrument (Applied Biosystems, ABI7500, Waltham, MA, USA). The comparative 2^(−ΔΔCT)^ method was used for relative quantification, with data representing the mean of three technical replicates. All primers used in this study were listed in Table S8.

For the fungal biomass, three representative maize leaves infected by pathogens with disease lesions were selected. A 4 cm-length leaf segment containing lesions was excised for extracting genomic DNA using the cetyltrimethylammonium bromide (CTAB) method. Relative fungal biomass was measured by DNA-based quantitative PCR (qPCR) based on threshold cycle (Ct) values of the *C. graminicola* gene *CgH3* and the maize reference gene *ZmACTRT*, calculated as 2^[CT(ZmACTRT)−CT(CgH3)]^.

### 4.11. Bioinformatics Analysis

Venn diagrams were generated using the online tool available at http://www.interactivenn.net/ (accessed on 7 May 2021). Heatmaps were created using Multiple Experiment Viewer (MeV) software (MeV 4.9.0). Principal component analysis (PCA) was performed and visualized using the vegan and ggplot2 packages (version 4.0.1) in R studio (version 4.3.0).

## 5. Conclusions

In this study, we identified CgMas2 as a secreted protein and a virulence factor essential for the full pathogenicity of *C. graminicola*. We found that deletion of *CgMAS2* impaired invasive hyphal growth, enhanced ROS accumulation, and reduced the infection capability of the fungus. Transcriptome analysis further revealed that CgMas2 likely manipulates plant hormone signaling and defense compound biosynthesis to promote necrotrophic infection. Therefore, our study provides new insights into the role of CgMas2, demonstrating that it functions not only in biotrophic invasive hyphae growth but may also modulate the ethylene signaling pathway and defense metabolite formation to facilitate fungal infection during the necrotrophic stage.

## Data Availability

The original contributions presented in this study are included in the article/Supplementary Materials. Further inquiries can be directed to the corresponding authors.

## Supplementary Materials

The following supporting information can be downloaded at https://www.mdpi.com/article/10.3390/ijms27020845/s1.

## Abbreviations

Hpi	hours post inoculation
Dpi	days post inoculation
IH	invasive hyphae
ET	ethylene
AUX	auxin
DEGs	differentially expressed genes
uDGs	upregulated differentially expressed genes
dDGs	downregulated differentially expressed genes
PCA	principal component analysis
FPKM	fragments per kilobase per million mapped reads
Go	Gene Ontology
KEGG	Kyoto Encyclopedia of Genes and Genomes
