# Supplementary material for: The Candidate Effector Cgmas2 Orchestrates Biphasic Infection of Colletotrichum graminicola in Maize by Coordinating Invasive Growth and Suppressing Host Immunity
Source: Int J Mol Sci. 2026 Jan 14;27(2):845. doi: 10.3390/ijms27020845 (PMC12840753; doi:10.3390/ijms27020845)
Supplement: Supplementary file 1 [file ijms-27-00845-s001.zip › Figure S1.pdf]

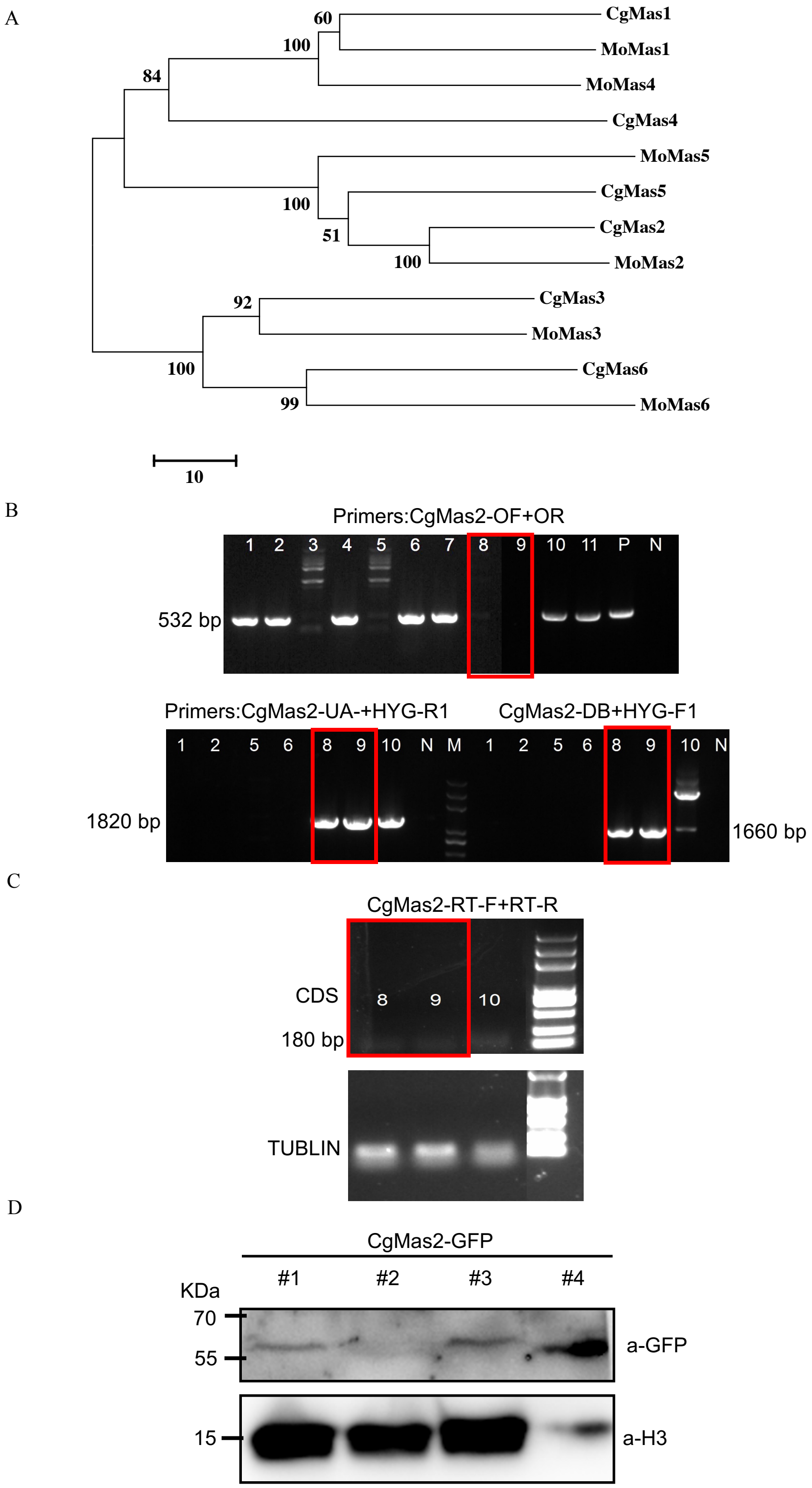

Figure S1. Identification of *CgMAS2* knockout mutants. (**A**) A phylogenetic tree of Mas proteins from *C. graminicola* and *M. oryzae* was constructed using the neighbor-joining method in MEGA7 with 1000 bootstrap replicates; all other parameters were set to default. The scale bar indicates branch length. (**B**) Screening of candidate deletion mutants by PCR using three different primer pairs. Primer names are indicated above the images, and expected fragment sizes are marked beside the bands. (**C**) RT-PCR analysis further confirming the knockout mutants. The red rectangles indicate the positive deletion mutants. (**D**) Western blot analysis of CgMas2-GFP expression in complementation strains using an anti-GFP antibody. Histone H3 was used as a loading control.
