# Supplementary material for: The Candidate Effector Cgmas2 Orchestrates Biphasic Infection of Colletotrichum graminicola in Maize by Coordinating Invasive Growth and Suppressing Host Immunity
Source: Int J Mol Sci. 2026 Jan 14;27(2):845. doi: 10.3390/ijms27020845 (PMC12840753; doi:10.3390/ijms27020845)
Supplement: Supplementary file 1 [file ijms-27-00845-s001.zip › Figure S2.pdf]

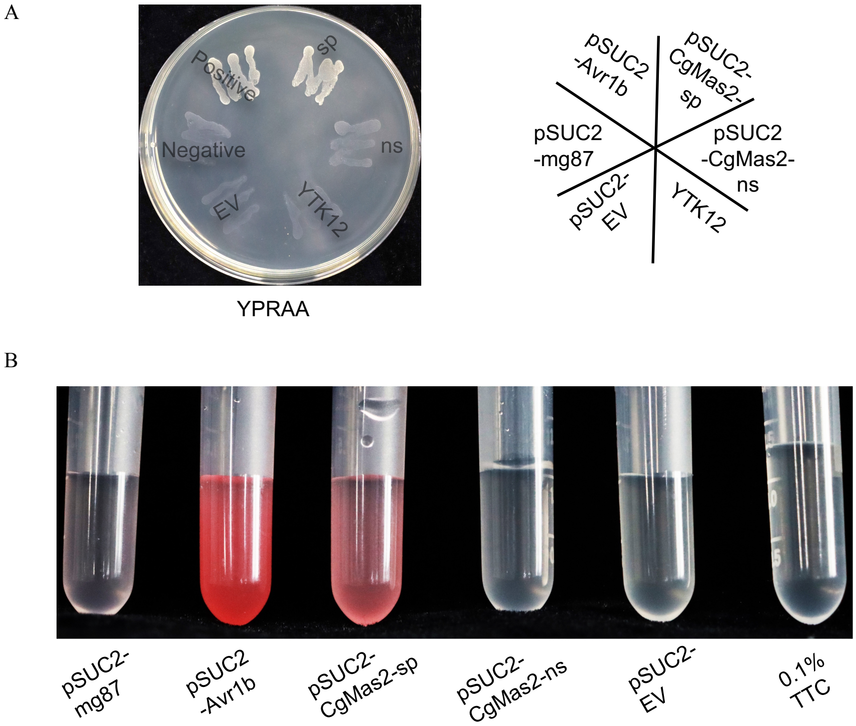

Figure S2. Cgmas2 possesses a functional signal peptide. (**A**) The secretion function of the CgMas2 signal peptide was validated using a yeast signal trap assay. The sequences encoding the CgMas2 signal peptide (CgMas2-sp) and a truncated version without the signal peptide (CgMas2-ns) were fused into the pSUC2 vector and transformed into the yeast strain YTK12. Avr1b and mg87 were used as positive and negative controls, respectively. (**B**) TTC assay further confirms the functionality of the CgMas2 signal peptide. All experiments were performed with three biological replicates, showing consistent results.
