# Supplementary material for: The Candidate Effector Cgmas2 Orchestrates Biphasic Infection of Colletotrichum graminicola in Maize by Coordinating Invasive Growth and Suppressing Host Immunity
Source: Int J Mol Sci. 2026 Jan 14;27(2):845. doi: 10.3390/ijms27020845 (PMC12840753; doi:10.3390/ijms27020845)
Supplement: Supplementary file 1 [file ijms-27-00845-s001.zip › Figure S3.pdf]

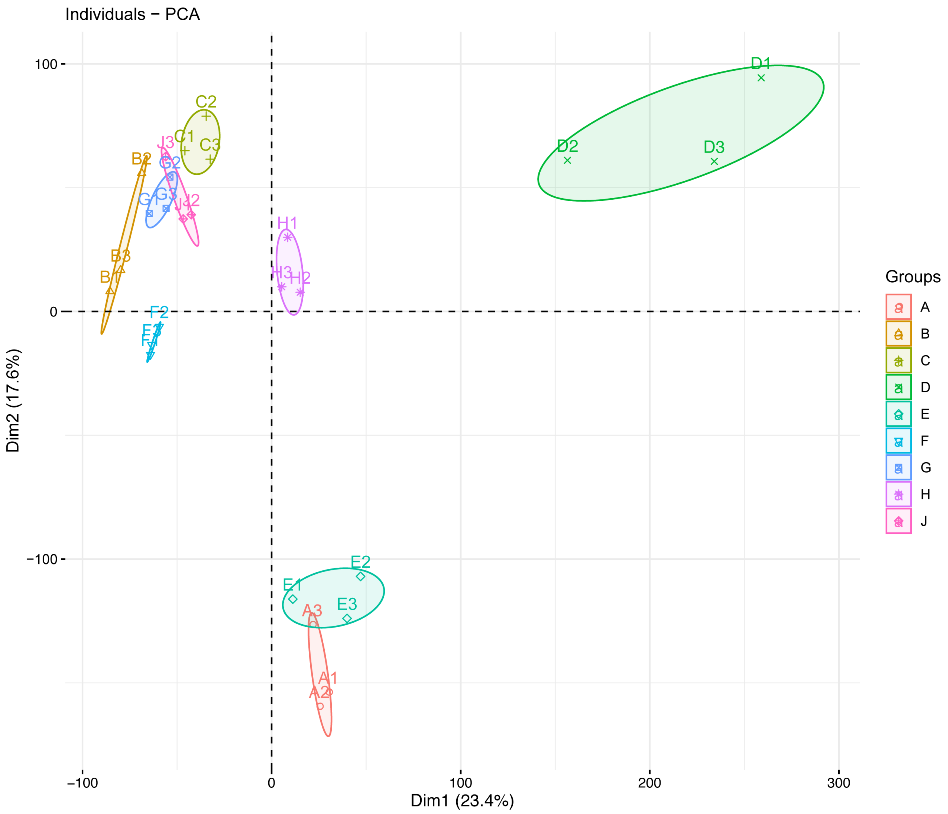

Figure S3. Principal component analysis (PCA) of RNA-seq data. The plot shows consistent clustering among three biological replicates for each treatment (inoculated vs. control) across four time points (24, 40, 60, and 96 hpi). A-D correspond to CgM2 at 24, 40, 60, and 96 hpi; E-H represent *ΔCgmas2* at the same time points; J indicates H_2O-treated controls.
