# Supplementary material for: The Candidate Effector Cgmas2 Orchestrates Biphasic Infection of Colletotrichum graminicola in Maize by Coordinating Invasive Growth and Suppressing Host Immunity
Source: Int J Mol Sci. 2026 Jan 14;27(2):845. doi: 10.3390/ijms27020845 (PMC12840753; doi:10.3390/ijms27020845)
Supplement: Supplementary file 1 [file ijms-27-00845-s001.zip › Figure S4.pdf]

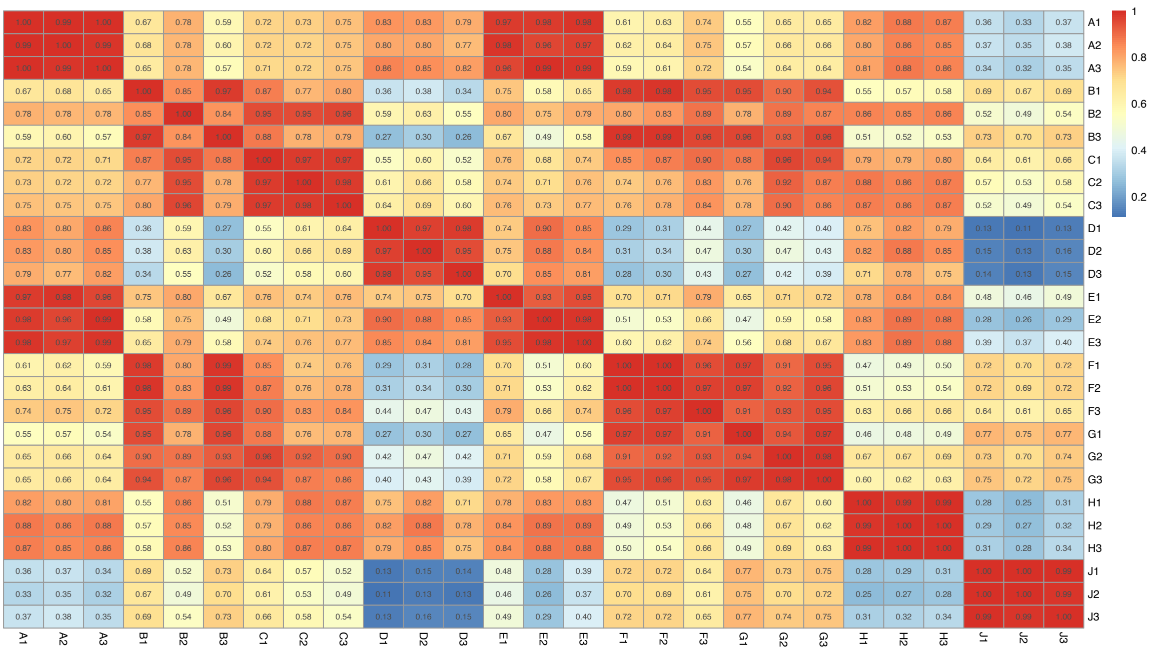


**Figure S4.** Pearson correlation matrix across samples. Red and blue shades indicate high and low correlation coefficients, respectively, with the color intensity corresponding to R² values. Labels A-D represent CgM2 samples at 24, 40, 60, and 96 hpi; E-H represent *ΔCgmas2* samples at the corresponding time points; J represents the 0 hpi mock control. Each condition includes three biological replicates.
