# Supplementary material for: The Candidate Effector Cgmas2 Orchestrates Biphasic Infection of Colletotrichum graminicola in Maize by Coordinating Invasive Growth and Suppressing Host Immunity
Source: Int J Mol Sci. 2026 Jan 14;27(2):845. doi: 10.3390/ijms27020845 (PMC12840753; doi:10.3390/ijms27020845)
Supplement: Supplementary file 1 [file ijms-27-00845-s001.zip › Figure S5.pdf]

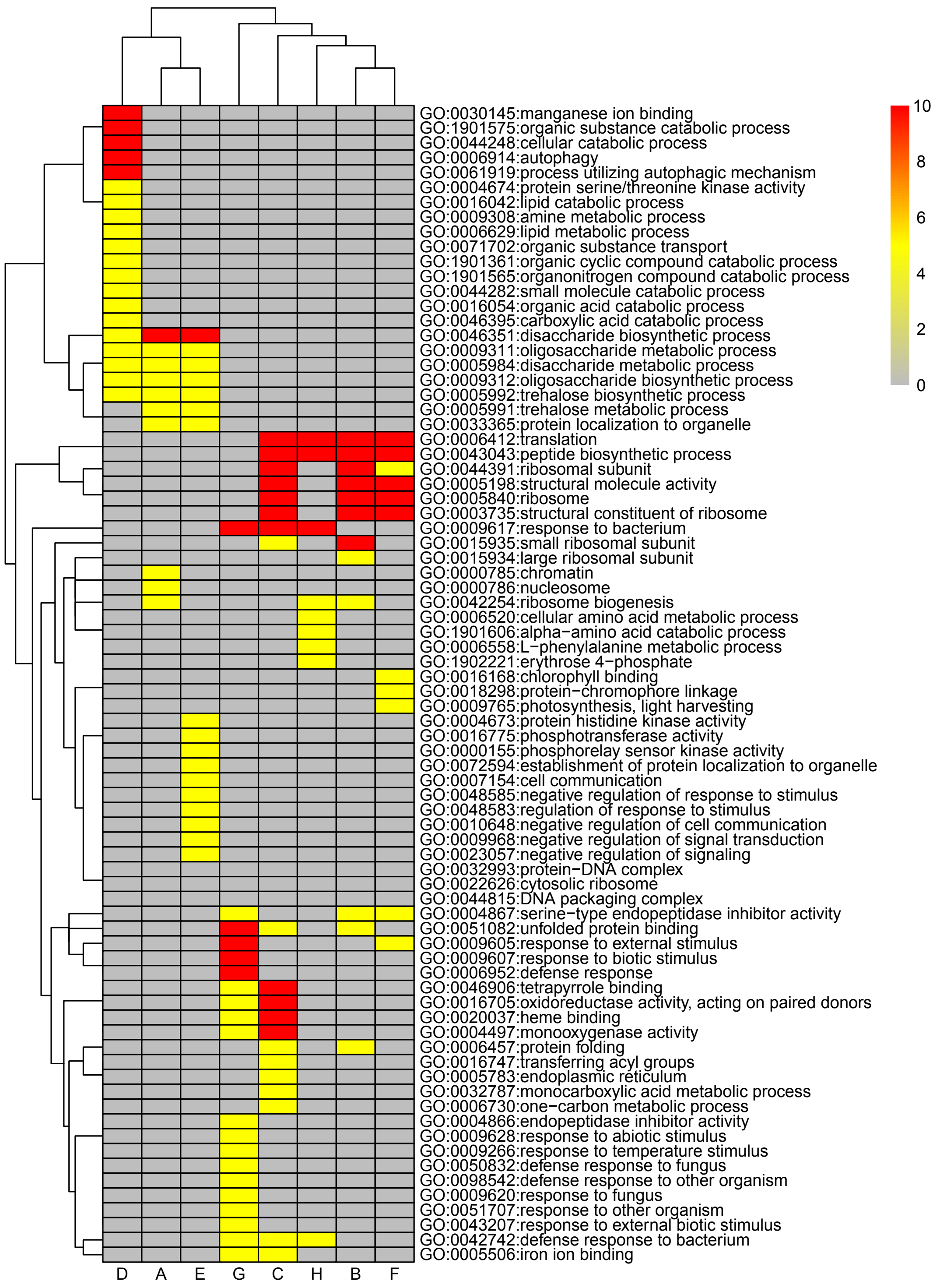

Figure S5. Top 20 (or all, if ≤20) enriched GO terms for up-regulated DEGs in maize plants infected with *C. graminicola*. Panels A-D correspond to CgM2 infections at 24, 40, 60, and 96 hpi; E-H represent *ΔCgmas2* infections at the same time points.
