# Supplementary material for: The Candidate Effector Cgmas2 Orchestrates Biphasic Infection of Colletotrichum graminicola in Maize by Coordinating Invasive Growth and Suppressing Host Immunity
Source: Int J Mol Sci. 2026 Jan 14;27(2):845. doi: 10.3390/ijms27020845 (PMC12840753; doi:10.3390/ijms27020845)
Supplement: Supplementary file 1 [file ijms-27-00845-s001.zip › Figure S6.pdf]

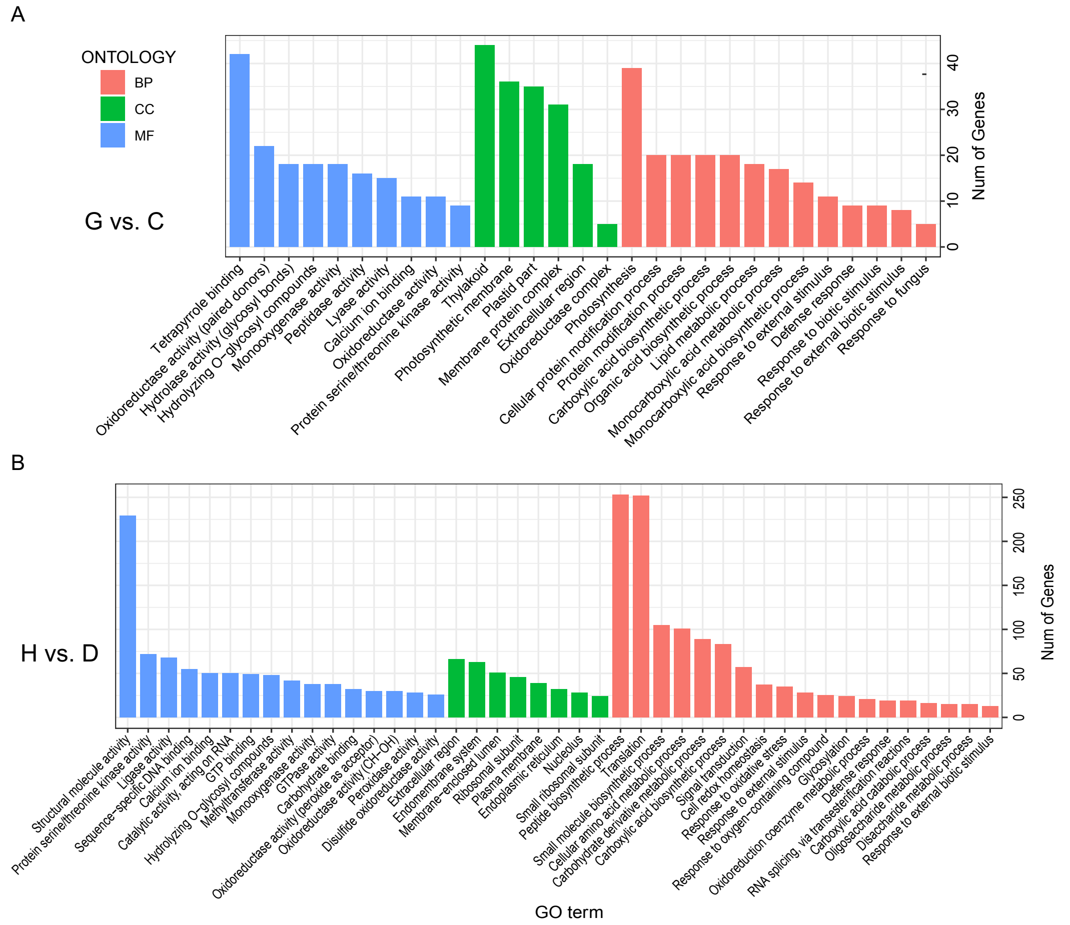

Figure S6. GO term enrichment analysis of DEGs from G vs. C and H vs. D comparisons during late infection stages. (**A**, **B**) Bar charts show GO terms enriched in biological process (BP), cellular component (CC), and molecular function (MF) categories for CgM2 vs. *ΔCgmas2* at 60 hpi (G vs. C) and 96 hpi (H vs. D).
