# Supplementary material for: The Candidate Effector Cgmas2 Orchestrates Biphasic Infection of Colletotrichum graminicola in Maize by Coordinating Invasive Growth and Suppressing Host Immunity
Source: Int J Mol Sci. 2026 Jan 14;27(2):845. doi: 10.3390/ijms27020845 (PMC12840753; doi:10.3390/ijms27020845)
Supplement: Supplementary file 1 [file ijms-27-00845-s001.zip › Figure S8.pdf]

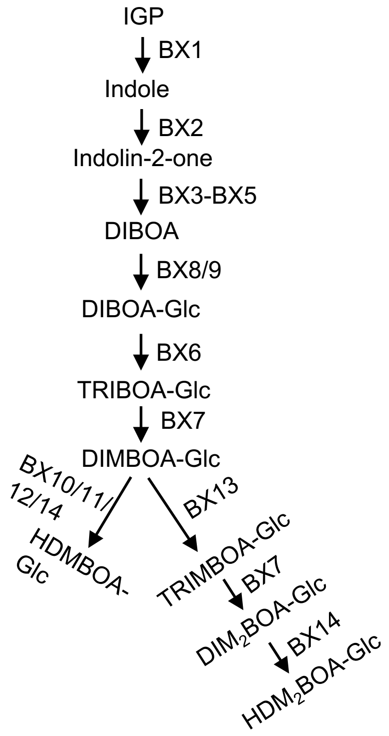

Figure S8. Benzoxazinoid biosynthesis pathways in maize. Indole is made by BX1 as the first step in benzoxazinoid biosynthesis. This indole is then converted into indolin-2- one by the indole-2-monooxygenase (BX2 - benzoxazinone synthesis 2). Mechanistically diverse BX enzymes catalyze the subsequent steps in benzoxazinoid production making a range of these glycosylated hydroxamic acids including DIMBOA-Glc (2,4-dihydroxy-7-methoxy-2H- 1,4-benzoxazin-3(4H)-one-b-D-glucopyranose), HDMBOA-Glc (2- hydroxy-4,7-dimethoxy-1,4-benzoxazin-3-one-b-D-glucopyranose), and HDM2BOA-Glc (2-2-hydroxy-4,7,8-trimethoxy-1,4-benzoxazin-3-one-b- D-glucopyranose),
