# Supplementary material for: The Candidate Effector Cgmas2 Orchestrates Biphasic Infection of Colletotrichum graminicola in Maize by Coordinating Invasive Growth and Suppressing Host Immunity
Source: Int J Mol Sci. 2026 Jan 14;27(2):845. doi: 10.3390/ijms27020845 (PMC12840753; doi:10.3390/ijms27020845)
Supplement: Supplementary file 1 [file ijms-27-00845-s001.zip › Table S2 Sequencing metrics of the 27 RNA-seq libraries.docx]

Table S2: Sequencing metrics of the 27 RNA-seq libraries.

| Description | Total clean reads | plant | |  | |  |  | | |
| --- | --- | --- | --- | --- | --- | --- | --- | --- | --- |
|  |  | total mapped | mapping rate | | Q20 | | | Q30 | GC content |
| CgM2 24 h (A1) | 103372852 | 94156827 | 0.910846757 | | 97.34 | | | 92.82 | 54.45 |
| CgM2 24 h (A2) | 81951128 | 74686274 | 0.911351385 | | 97.28 | | | 92.69 | 54.55 |
| CgM2 24 h (A3) | 88815634 | 80296508 | 0.904080784 | | 97.44 | | | 92.99 | 54.64 |
| CgM2 40 h (B1) | 75536438 | 70541490 | 0.933873662 | | 97.90 | | | 94.09 | 55.63 |
| CgM2 40 h (B2) | 69974534 | 65206551 | 0.931861168 | | 97.71 | | | 93.60 | 55.09 |
| CgM2 40 h (B3) | 76862838 | 71907531 | 0.935530523 | | 98.02 | | | 94.37 | 55.64 |
| CgM2 60 h (C1) | 76781178 | 71977125 | 0.937431892 | | 97.88 | | | 94.02 | 55.50 |
| CgM2 60 h (C2) | 73631562 | 69186586 | 0.939632192 | | 98.02 | | | 94.37 | 55.54 |
| CgM2 60 h (C3) | 72514532 | 67886995 | 0.936184695 | | 97.80 | | | 93.84 | 55.78 |
| CgM2 96 h (D1) | 72365316 | 60015875 | 0.829345857 | | 97.60 | | | 93.34 | 55.94 |
| CgM2 96 h (D2) | 72343698 | 66206573 | 0.91516711 | | 97.57 | | | 93.32 | 55.57 |
| CgM2 96 h (D3) | 81456984 | 74954621 | 0.920174273 | | 97.76 | | | 93.75 | 56.03 |
| *∆Cgmas2* 24 h (E1) | 75014406 | 68986115 | 0.919638223 | | 97.58 | | | 93.32 | 55.27 |
| *∆Cgmas2* 24 h (E2) | 69070000 | 63170482 | 0.914586391 | | 97.76 | | | 93.73 | 54.81 |
| *∆Cgmas2* 24 h (E3) | 72877790 | 64003527 | 0.878230899 | | 97.79 | | | 93.81 | 55.12 |
| *∆Cgmas2* 40 h (F1) | 78727950 | 72936515 | 0.926437371 | | 97.65 | | | 93.46 | 55.81 |
| *∆Cgmas2* 40 h (F2) | 75916178 | 69047818 | 0.909527058 | | 96.94 | | | 91.94 | 56.09 |
| *∆Cgmas2* 40 h (F3) | 65831612 | 61031889 | 0.927090909 | | 97.74 | | | 93.71 | 55.24 |
| *∆Cgmas2* 60 h (G1) | 71357752 | 65360653 | 0.915957288 | | 97.74 | | | 93.68 | 55.89 |
| *∆Cgmas2* 60 h (G2) | 73613202 | 68728571 | 0.933644633 | | 97.58 | | | 93.33 | 55.47 |
| *∆Cgmas2* 60 h (G3) | 72665576 | 67632527 | 0.930736818 | | 97.50 | | | 93.14 | 55.66 |
| *∆Cgmas2* 96 h (H1) | 71442698 | 66503728 | 0.930868092 | | 97.68 | | | 93.58 | 54.81 |
| *∆Cgmas2* 96 h (H2) | 73059552 | 67393384 | 0.922444529 | | 97.36 | | | 92.82 | 54.67 |
| *∆Cgmas2* 96 h (H3) | 70917500 | 65988139 | 0.930491613 | | 97.69 | | | 93.57 | 54.42 |
| H_2_O (J1) | 78931414 | 74314868 | 0.941511931 | | 97.89 | | | 94.03 | 56.59 |
| H_2_O (J2) | 80692216 | 75587363 | 0.936736736 | | 97.57 | | | 93.28 | 56.54 |
| H_2_O (J3) | 76486216 | 71904442 | 0.940096736 | | 97.80 | | | 93.74 | 56.40 |

A: CgM2 24 h, B: CgM2 40 h, C: CgM2 60 h, D: CgM2 96 h, E: *∆Cgmas2* 24 h; F: *∆Cgmas2* 40 h; G: *∆Cgmas2* 60 h; H: *∆Cgmas2* 96 h, J: H_2_O.
