# Supplementary material for: The Candidate Effector Cgmas2 Orchestrates Biphasic Infection of Colletotrichum graminicola in Maize by Coordinating Invasive Growth and Suppressing Host Immunity
Source: Int J Mol Sci. 2026 Jan 14;27(2):845. doi: 10.3390/ijms27020845 (PMC12840753; doi:10.3390/ijms27020845)
Supplement: Supplementary file 1 [file ijms-27-00845-s001.zip › Table S6 KEGG pathway enrichment of DEGs from C. graminicola -infected maize plants.doc]

Table S6. KEGG pathway enrichment of DEGs from *C. graminicola* -infected maize plants.

| ID | Pathway | p. adjusted | | | | | | | | |
| --- | --- | --- | --- | --- | --- | --- | --- | --- | --- | --- |
|  |  | CgM2 | | | | | *∆Cgmas2* | | | |
|  |  | A (24h) | | B (40h) | C (60h) | D (96h) | E (24h) | F (40h) | G (60h) | H (96h) |
| UP-REGULATED ENRICHMENT KEGG PATHWAYS | | | | | | | | | | |
| zma00280 | Valine, leucine and isoleucine degradation | 7.15e-05 |  | | 0.020052 | 1.07e-09 | 3.64e-07 |  |  | 1.89e-05 |
| zma04712 | Circadian rhythm - plant | 7.15e-05 |  | |  |  | 6.83e-05 |  |  |  |
| zma03008 | Ribosome biogenesis in eukaryotes | 7.15e-05 |  | |  |  |  |  |  |  |
| zma03040 | Spliceosome | 0.00037 |  | |  |  |  |  |  |  |
| zma03010 | Ribosome |  | 1.34e-45 | | 1.07e-18 |  |  | 5.75e-10 |  | 3.22e-44 |
| zma00945 | Stilbenoid, diarylheptanoid and gingerol biosynthesis |  |  | | 5.74e-06 |  |  |  | 0.010675 | 0.003100 |
| zma04141 | Protein processing in endoplasmic reticulum | 0.023820 | 6.23e-05 | | 2.37e-05 | 0.036623 | 0.006158 |  | 0.000885 |  |
| zma00941 | Flavonoid biosynthesis |  |  | | 3.33e-05 |  |  |  | 0.023707 | 0.004046 |
| zma00640 | Propanoate metabolism |  |  | | 0.000775 | 1.63e-06 | 0.027963 | 0.039002 | 0.023707 | 0.000295 |
| zma00480 | Glutathione metabolism |  |  | | 0.000775 |  |  |  |  |  |
| zma00940 | Phenylpropanoid biosynthesis |  |  | | 0.001840 |  |  |  | 0.023707 |  |
| zma04016 | MAPK signaling pathway - plant | 0.028906 |  | |  | 0.000110 | 9.01e-05 |  | 0.035610 | 0.005938 |
| zma04146 | Peroxisome | 0.028906 |  | |  |  | 0.020569 |  |  |  |
| zma00592 | alpha-Linolenic acid metabolism |  |  | |  | 5.62e-06 | 0.036218 |  | 0.020621 | 0.004061 |
| zma04626 | Plant-pathogen interaction |  |  | | 0.002030 | 0.000200 |  |  | 0.026283 | 0.005316 |
| zma00071 | Fatty acid degradation |  |  | | 0.004903 | 2.70e-07 | 0.017576 |  |  | 0.000450 |
| zma00270 | Cysteine and methionine metabolism |  |  | |  |  |  |  |  | 0.004046 |
| zma01212 | Fatty acid metabolism |  |  | |  | 9.04e-05 |  |  |  |  |
| zma00410 | beta-Alanine metabolism |  |  | |  | 0.000110 |  |  |  |  |
| zma04136 | Autophagy - other |  |  | |  | 0.000258 |  |  |  |  |
| zma00052 | Galactose metabolism |  |  | |  | 0.002896 | 0.017576 |  |  |  |
| zma00500 | Starch and sucrose metabolism |  |  | |  |  | 0.020978 |  |  |  |
| zma00196 | Photosynthesis - antenna proteins |  |  | |  |  |  | 0.010201 |  |  |
| zma00904 | Diterpenoid biosynthesis |  |  | |  |  |  |  | 0.020621 |  |
| zma00520 | Amino sugar and nucleotide sugar metabolism |  |  | |  |  |  |  | 0.026283 |  |
| DOWN-REGULATED ENRICHMENT KEGG PATHWAYS | | | | | | | | | | |
| zma00710 | Carbon fixation in photosynthetic organisms | 3.34e-11 | | 3.08e-10 | 1.18e-09 | 5.02e-07 | 1.76e-10 | 4.25e-06 | 2.02e-07 | 5.49e-09 |
| zma01200 | Carbon metabolism | 1.37e-10 | | 3.05e-05 | 4.22e-05 | 2.78e-07 | 2.92e-09 | 0.000286 | 1.88e-05 | 8.50e-06 |
| zma00195 | Photosynthesis | 4.07e-10 | | 3.08e-10 | 9.23e-27 | 1.12e-17 | 7.52e-11 | 4.25e-06 | 9.67e-09 | 2.05e-22 |
| zma00402 | Benzoxazinoid biosynthesis | 5.32e-07 | | 0.000100 | 2.96e-05 | 3.99e-05 | 2.65e-07 | 1.69e-05 | 9.94e-05 | 4.54e-07 |
| zma01240 | Biosynthesis of cofactors | 5.32e-07 | | 0.007920 | 0.000273 | 3.44e-05 | 1.17e-06 | 5.92e-05 | 4.58e-07 | 0.001479 |
| zma01230 | Biosynthesis of amino acids | 2.78e-06 | |  |  |  |  |  |  |  |
| zma00010 | Glycolysis / Gluconeogenesis | 1.53e-05 | | 0.004681 | 0.013389 |  | 3.02e-05 | 0.000902 | 0.000551 |  |
| zma00620 | Pyruvate metabolism | 1.53e-05 | |  |  | 0.000450 | 3.02e-05 |  |  |  |
| zma00500 | Starch and sucrose metabolism |  | | 0.000525 | 0.013133 |  | 3.40e-05 | 0.001342 |  | 0.000295 |
| zma00910 | Nitrogen metabolism |  | | 0.007920 |  |  |  | 0.001342 | 0.009870 |  |
| zma00030 | Pentose phosphate pathway |  | | 0.009002 |  |  |  |  |  |  |
| zma00860 | Porphyrin and chlorophyll metabolism |  | |  | 7.55e-08 | 2.14e-06 | 0.000242 |  | 3.71e-05 | 1.02e-08 |
| zma00196 | Photosynthesis - antenna proteins | 9.32e-05 | |  | 1.03e-06 | 6.54e-11 | 3.40e-05 |  |  | 6.52e-14 |
| zma00360 | Phenylalanine metabolism |  | |  |  |  |  | 0.002088 |  |  |
| zma00130 | Ubiquinone and other terpenoid-quinone biosynthesis |  | |  |  |  |  | 0.003626 | 0.002006 | 0.001254 |
| zma00051 | Fructose and mannose metabolism |  | | 0.010671 | 0.015401 |  |  |  | 0.002006 |  |
| zma03010 | Ribosome |  | |  |  | 5.02e-07 |  |  |  |  |
| zma00630 | Glyoxylate and dicarboxylate metabolism |  | |  |  | 0.000202 |  |  |  | 0.001370 |
| zma00020 | Citrate cycle (TCA cycle) | 0.000139 | |  |  |  |  |  |  |  |
