# Supplementary material for: The Candidate Effector Cgmas2 Orchestrates Biphasic Infection of Colletotrichum graminicola in Maize by Coordinating Invasive Growth and Suppressing Host Immunity
Source: Int J Mol Sci. 2026 Jan 14;27(2):845. doi: 10.3390/ijms27020845 (PMC12840753; doi:10.3390/ijms27020845)
Supplement: Supplementary file 1 [file ijms-27-00845-s001.zip › Table S7 the DEGs of H Vs. D related to ethylene pathway.doc]

Table S7. The DEGs of *ΔCgmas2* Vs. CgM2 96 hpi related to ethylene pathway

| Gene ID | Gene annotation | | Log_2_ fold change | |
| --- | --- | --- | --- | --- |
| Zm00001d016616 | | Ethylene responsive transcription factor ERF115 | | -2.30 |
| Zm00001d019216 | | Ethylene responsive transcription factor ERF118 | | -3.02 |
| Zm00001d018305 | | Ethylene responsive transcription factor RAP2-3 | | -2.12 |
| Zm00001d053859 | | Ethylene responsive transcription factor 3 | | -2.52 |
| Zm00001d043491 | | Ethylene responsive transcription factor 2 | | -2.17 |
| Zm00001d020267 | | Ethylene responsive transcription factor ERF056 | | -1.29 |
| Zm00001d038852 | | Reversion to ethylene sensitivity1 like3 | | -1.32 |
| Zm00001d043205 | | Ethylene responsive transcription factor 4 | | -1.14 |
| Zm00001d026191 | | Ethylene responsive transcription factor 1A | | -1.04 |
| Zm00001d053642 | | EIN3-binding_F-box_protein_1 | | 2.48 |
| Zm00001d016535 | | Ethylene responsive transcription factor like protein | | -1.21 |
| Zm00001d021089 | | Ethylene responsive transcription factor ERF112 | | -4.38 |
| Zm00001d005798 | | Ethylene responsive transcription factor RAP2-2 | | -4.82 |
| Zm00001d020540 | | AP2 like ethylene responsive transcription factor | | -3.27 |
| Zm00001d030513 | | Ethylene responsive transcription factor ERF055 | | -2.44 |
| Zm00001d020939 | | Ethylene insensitive3 like 3 protein | | -1.06 |
| Zm00001d020595 | | Ethylene response factor | | 2.25 |
| Zm00001d019475 | | Ethylene responsive transcription factor 1B | | 2.91 |
