# Supplementary material for: The Candidate Effector Cgmas2 Orchestrates Biphasic Infection of Colletotrichum graminicola in Maize by Coordinating Invasive Growth and Suppressing Host Immunity
Source: Int J Mol Sci. 2026 Jan 14;27(2):845. doi: 10.3390/ijms27020845 (PMC12840753; doi:10.3390/ijms27020845)
Supplement: Supplementary file 1 [file ijms-27-00845-s001.zip › Table S8 Oligonucleotide primers were used in this study.doc]

Table S8. Oligonucleotide primers were used in this study.

| Gene | Gene ID | F (from 5’ to 3’) | R (from 5’ to 3’) |
| --- | --- | --- | --- |
| CgMas2-up | GLRG_03323 | AGATTCTGTAGGTGGAGATGGGA | ACCTCCACTAGCTCCAGCCAAGCGTGTCGGAAAGAGAGGCA |
| CgMas2-dn | GLRG_03323 | GAATAGAGTAGATGCCGACCGGGACCAGGCAACAAGGAAATCAA | TGGGGAACCATCTGTGTAAGTG |
| CgMas2-OF/R | GLRG_03323 | CGCTGCTGGCAACACGA | GTTGAACTCAATGGCACGCTT |
| CgMas2-UA/DB | GLRG_03323 | GGCTTCTTGAGATCGTCGTGT | GCCAGGATAGCAGGAATCAGAG |
| HYG-F1/R1 | Hygromycin B | CGTTGCAAGACCTGCCTGAA | GGATGCCTCCGCTCGAAGTA |
| CgMas2-RT-F/R | GLRG_03323 | AGGAAATGATGGCTCGTCG | TCTTGGTTGACCTGGTGGAA |
| CgMas2-SP-F/R | GLRG_03323 | CGGAATTTTAATTAAGAATTCATGAAGTACAGCTCCAC | CACTATAGGGAGAACCTCGAGGGCGAGGACGTTGGTG |
| CgMas2-NS-F/R | GLRG_03323 | CGGAATTTTAATTAAGAATTCCACGGTGTCATCGACAG | CACTATAGGGAGAACCTCGAGGGCCTTGAAAGCGCGT |
| CgMas2-RP27 | GLRG_03323 | CCAAGCTTATGAAGTACAGCTCCACC | ACCATTCTAGAACTAGTGGATCCGGCCTTGAAAGCGCGT |
| Benzosys3 | Zm00001d048702 | CGCACCCAGGACCACATCTT | ACAGGAGATCGCTCAGGTCC |
| Benzosys4 | Zm00001d048703 | CTTCTGGAGCTTGGCCAGGA | CTCTGAAGACTTCGTGCACC |
| Benzosys5 | Zm00001d048705 | CAACACCTGCGACGTGGCCT | CTCACCACGTCGTTGGTGTA |
| ERF2 | Zm00001d043491 | ATGAGCACGTACATATCTCT | CCTTCAGCGTAGGCAACGA |
| ERF3 | Zm00001d053859 | CTGCTGCAAGGAGCACCAGA | CGTTTCAGACTGTCCTGTGT |
| ERF56 | Zm00001d020267 | GAGGAGCTGATGAGAGCACT | GGATGTACTGCATCTGGGAA |
| ERF112 | Zm00001d021089 | ATGCATGGGCAGAGAGGCGT | CACGCCCCTGTACTGATGT |
| ERF115 | Zm00001d016616 | ATGGCGTACTACGACGACGTG | CCACGGTAGTGCCTCATCGT |
| ERF118 | Zm00001d019216 | ATGGACCCTAGCCTGAAGGA | CAGCACGCGGCTGCGCTTCA |
